# Correction: Modeling wind erosion susceptibility of Eastern Iran using machine learning

**DOI:** 10.1371/journal.pone.0360012

**Published:** 2026-10-06

**Authors:** The PLOS One Staff

## Notice of Republication

This article was republished on September 23, 2026 to correct an error in the fourth author’s name. The publisher apologizes for the errors. Please download this article again to view the correct version. The originally published, uncorrected article and the republished, corrected articles are provided here for reference.

## Supporting information

S1 File Originally published, uncorrected article. (PDF)

S2 File Republished, corrected article. (PDF)
