# Supplementary material for: Modeling wind erosion susceptibility of Eastern Iran using machine learning
Source: PLoS One. 2026 Aug 6;21(8):e0354288. doi: 10.1371/journal.pone.0354288 (PMC13446583; doi:10.1371/journal.pone.0354288)
Supplement: Supplementary file 1 [file pone.0354288.s001.docx]

Table S1: List of predictive variables used in wind erosion susceptibility modeling.

| **Category** | **Variable name** | | **Description** | **Abbreviation** | **Units** | H1 | H2 | H3 |
| --- | --- | --- | --- | --- | --- | --- | --- | --- |
| Bioclimatic^*^ | Annual Mean temperature | | The monthly average temperature | BIO 1 | (◦C) | Reject | Reject | Reject |
|  | Mean diurnal temperature | | Average monthly (max temp – min temp) | BIO 2 | (◦C) | Accept | Accept | Accept |
|  | Isothermality | | (BIO2/BIO7) (×100) | BIO 3 | Percent | Accept | Accept | Accept |
|  | Temperature seasonality | | The amount of annual temperature variation calculated from the standard deviation of monthly temperature averages×100 | BIO 4 | (◦C) | Accept | Accept | Accept |
|  | Maximum temperature of the warmest month | | The highest monthly temperature that has been recorded in a certain year | BIO 5 | (◦C) | Reject | Reject | Reject |
|  | Minimum Temperature of the Coldest Month | | The occurrence of the lowest monthly temperature in a given year | BIO 6 | (◦C) | Reject | Reject | Reject |
|  | Temperature Annual Range | | Temperature variation over a given period (BIO5-BIO6) | BIO 7 | (◦C) | Reject | Reject | Accept |
|  | Mean temperature of the wettest quarter | | The average temperatures experienced during the wettest quarter | BIO 8 | (◦C) | Accept | Accept | Accept |
|  | Mean temperature of the driest quarter | | The average temperatures experienced during the driest quarter | BIO 9 | (◦C) | Reject | Reject | Reject |
|  | Mean temperature of the warmest quarter | | The average temperatures experienced during the hottest quarter | BIO 10 | (◦C) | Reject | Reject | Reject |
|  | Mean temperature of the coldest quarter | | The average temperatures in the coldest quarter | BIO 11 | (◦C) | Reject | Reject | Accept |
|  | Annual precipitation | | This is the sum of all total monthly precipitation values | BIO 12 | mm | Accept | Reject | Reject |
|  | Precipitation of the wettest month | | The total amount of precipitation experienced in the wettest month | BIO 13 | mm | Reject | Reject | Reject |
|  | Precipitation of the driest month | | The total amount of precipitation experienced in the driest month | BIO 14 | mm | Accept | Accept | Accept |
|  | Precipitation seasonality | | The monthly total precipitation standard deviation from the monthly total precipitation means | BIO 15 | Percent | Accept | Accept | Accept |
|  | Precipitation of the wettest quarter | | The total amount of precipitation experienced during the wettest quarter | BIO 16 | mm | Reject | Reject | Reject |
|  | Precipitation of the driest quarter | | The overall amount of precipitation experienced during the driest quarter | BIO 17 | mm | Accept | Accept | Accept |
|  | Precipitation of the warmest quarter | | The total amount of precipitation that falls during the hottest | BIO 18 | mm | Reject | Reject | Accept |
|  | Precipitation of the Coldest Quarter | | The total amount of precipitation experienced during the coldest quarter | BIO 19 | mm | Reject | Reject | Reject |
| Wind^**^ | windspeed | | windspeed10m | | m/s | Accept | Accept | Accept |
|  |  |  | Windspeed50m | | m/s | Reject | Reject | Reject |
|  |  |  | windspeed100m | | m/s | Reject | Reject | Reject |
|  |  |  | windspeed150m | | m/s | Reject | Reject | Reject |
|  |  |  | Windspeed200m | | m/s | Reject | Reject | Reject |
| Edaphic^***^ | Physical properties | Bulk density | Bulk density in depth 0-5 centimeter | Bulk 0-5 | cg/cm^3^ | Accept | Accept | Accept |
|  |  | Bulk density | Bulk density in depth 5-15 centimeter | Bulk 5-15 | cg/cm^3^ | Accept | Accept | Accept |
|  |  | Bulk density | Bulk density in depth 15-30 centimeter | Bulk 15-30 | cg/cm^3^ | Accept | Accept | Accept |
|  |  | Bulk density | Bulk density in depth 30-60 centimeter | Bulk 30-60 | cg/cm^3^ | Accept | Accept | Reject |
|  |  | Bulk density | Bulk density in depth 60-100 centimeter | Bulk 60-100 | cg/cm^3^ | Reject | Reject | Reject |
|  |  | Sand | Sand in depth 0-5 centimeter | Sand 0-5 | g/kg | Accept | Accept | Accept |
|  |  | Sand | Sand in depth 5-15 centimeter | Sand 5-15 | g/kg | Reject | Reject | Reject |
|  |  | Sand | Sand in depth 15-30 centimeter | Sand 15-30 | g/kg | Reject | Reject | Reject |
|  |  | Sand | Sand in depth 30-60 centimeter | Sand 30-60 | g/kg | Reject | Reject | Reject |
|  |  | Sand | Sand in depth 30-60 centimeter | Sand 60-100 | g/kg | Reject | Reject | Reject |
|  |  | Silt | Silt in depth 0-5 centimeter | Silt 0-5 | g/kg | Accept | Accept | Accept |
|  |  | Silt | Silt in depth 5-15 centimeter | Silt 5-15 | g/kg | Reject | Reject | Reject |
|  |  | Silt | Silt in depth 15-30 centimeter | Silt 15-30 | g/kg | Reject | Reject | Reject |
|  |  | Silt | Silt in depth 30-60 centimeter | Silt 30-560 | g/kg | Reject | Reject | Reject |
|  |  | Silt | Silt in depth 60-100 centimeter | Silt 60-100 | g/kg | Reject | Reject | Reject |
|  |  | Clay content | Clay content in depth 0-5 centimeter | Clay 0-5 | g/kg | Accept | Accept | Accept |
|  |  | Clay content | Clay content in depth 5-15 centimeter | Clay 5-15 | g/kg | Reject | Reject | Reject |
|  |  | Clay content | Clay content in depth 15-30 centimeter | Clay 15-30 | g/kg | Reject | Reject | Reject |
|  |  | Clay content | Clay content in depth 30-60 centimeter | Clay 30-60 | g/kg | Reject | Reject | Reject |
|  |  | Clay content | Clay content in depth 60-100 centimeter | Clay 60-100 | g/kg | Reject | Reject | Reject |
|  |  | Coarse fragments | Coarse fragments in depth 0-5 centimeter | Coarse 0-5 | cm^3^/dm^3^ | Accept | Accept | Accept |
|  |  | Coarse fragments | Coarse fragments in depth 5-15 centimeter | Coarse 5-15 | cm^3^/dm^3^ | Reject | Reject | Reject |
|  |  | Coarse fragments | Coarse fragments in depth 15-30 centimeter | Coarse 15-30 | cm^3^/dm^3^ | Reject | Reject | Reject |
|  |  | Coarse fragments | Coarse fragments in depth 30-60 centimeter | Coarse 30-60 | cm^3^/dm^3^ | Reject | Reject | Reject |
|  |  | Coarse fragments | Coarse fragments in depth 60-100 centimeter | Coarse 60-100 | cm^3^/dm^3^ | Reject | Reject | Reject |
|  | Chemical properties | Nitrogen | Nitrogen in depth 0-5 centimeter | Nitrogen 0-5 | cg/kg | Accept | Accept | Accept |
|  |  | Nitrogen | Nitrogen in depth 5-15 centimeter | Nitrogen 5-15 | cg/kg | Accept | Accept | Accept |
|  |  | Nitrogen | Nitrogen in depth 15-30 centimeter | Nitrogen 15-30 | cg/kg | Accept | Accept | Accept |
|  |  | Nitrogen | Nitrogen in depth 30-60 centimeter | Nitrogen 30-60 | cg/kg | Accept | Accept | Accept |
|  |  | Nitrogen | Nitrogen in depth 60-100 centimeter | Nitrogen 60-100 | cg/kg | Accept | Accept | Accept |
|  |  | Soil organic carbon | Soil organic carbon in depth 0-5 centimeter | OC 0-5 | dg/kg | Accept | Accept | Accept |
|  |  | Soil organic carbon | Soil organic carbon in depth 5-15 centimeter | OC 5-15 | dg/kg | Accept | Accept | Accept |
|  |  | Soil organic carbon | Soil organic carbon in depth 15-30 centimeter | OC 15-30 | dg/kg | Accept | Accept | Accept |
|  |  | Soil organic carbon | Soil organic carbon in depth 30-60 centimeter | OC 30-60 | dg/kg | Accept | Accept | Accept |
|  |  | Soil organic carbon | Soil organic carbon in depth 60-100 centimeter | OC 60-100 | dg/kg | Accept | Accept | Accept |
|  |  | Soil pH water | Soil pH water in depth 0-5 centimeter | pHw 0-5 | pH * 10 | Accept | Accept | Accept |
|  |  | Soil pH water | Soil pH water in depth 5-15 centimeter | pHw 5-15 | pH * 10 | Reject | Reject | Reject |
|  |  | Soil pH water | Soil pH water in depth 15-30 centimeter | pHw 15-30 | pH * 10 | Reject | Reject | Reject |
|  |  | Soil pH water | Soil pH water in depth 30-60 centimeter | pHw 30-60 | pH * 10 | Reject | Reject | Reject |
|  |  | Soil pH water | Soil pH water in depth 60-100 centimeter | pHw 60-100 | pH * 10 | Reject | Reject | Reject |
|  |  | Cation exchange capacity | Cation exchange capacity (at pH 7) in depth 0-5 centimeter | CEC 0-5 | mmol(c)/kg | Accept | Accept | Accept |
|  |  | Cation exchange capacity | Cation exchange capacity (at pH 7) in depth 5-15 centimeter | CEC 5-15 | mmol(c)/kg | Reject | Reject | Reject |
|  |  | Cation exchange capacity | Cation exchange capacity (at pH 7) in depth 15-30 centimeter | CEC 15-30 | mmol(c)/kg | Reject | Reject | Reject |
|  |  | Cation exchange capacity | Cation exchange capacity (at pH 7) in depth 30-60 centimeter | CEC 30-60 | mmol(c)/kg | Reject | Reject | Reject |
|  |  | Cation exchange capacity | Cation exchange capacity (at pH 7) in depth 60-100 centimeter | CEC 60-100 | mmol(c)/kg | Reject | Reject | Reject |
|  |  | World Reference Base | World Reference Base (2006) Soil Groups | WRB 2006 | Unitless | Accept | Accept | Accept |
| Edaphic^****^ | index | | S | Salinity | Unitless | Accept | Accept | Reject |
|  |  |  | IS | Salinity Index | Unitless | Accept | Accept | Accept |
|  |  |  | NDSI | Normalized Difference Salinity Index | Unitless | Accept | Accept | Accept |
|  |  |  | BI | Brightness Index | Unitless | Accept | Accept | Accept |
|  |  |  | PD322 | Potential Different | Unitless | Accept | Accept | Accept |
|  |  |  | IPVI | Infrared Percentage Vegetation Index | Unitless | Accept | Accept | Accept |
|  |  |  | CRSI | Canopy Response Salinity Index | Unitless | Accept | Accept | Reject |
| Landuse^****^ | landuse | | landuse | | Unitless | Accept | Accept | Accept |
| LST^****^ | lst | | LST | Land Surface Temperature | Unitless | Accept | Accept | Accept |
| Geology^*****^ | geology | | Geology_epoch | | Unitless | Accept | Accept | Accept |
|  |  |  | Geology_age | | Unitless | Accept | Accept | Accept |
|  |  |  | geology | | Unitless | Accept | Accept | Accept |
| Vegetation^****^ | index | | NDVI | Normalized Differential Vegetation Index | Unitless | Accept | Accept | Accept |
|  | index | | NDBI | Normalized Differential Built-up Index | Unitless | Accept | Accept | Accept |
| Hydrologic^****^ | index | | NDMI | Normalized Differential Moisture Index | Unitless | Accept | Accept | Accept |
|  |  |  | NDWI | Normalized Differential water Index | Unitless | Accept | Accept | Accept |
| Topographic^*****^ | Altitude | | Altitude above sea level (obtained from optical sensors ASTER satellite, 90 m) | DEM | m | Accept | Accept | Accept |
|  | TWI | | Topographic wetness index (Produced from DEM) | TWI | Unitless | Accept | Accept | Accept |
|  | TRI | | Terrain Ruggedness Index | TRI | Unitless | Reject | Reject | Reject |
|  | Slope | | Percent change in that elevation over a certain distance | Slope | Degree | Accept | Accept | Accept |
| * <https://www.worldclim.org>  ** https://globalsolaratlas.info/download/iran  *** https://soilgrids.org/  **** Google Earth Engine  *****Iran Geological Organization  ****** www.earthexplorer.usgs.gov | | | | | | | | |
