# Supplementary material for: Modeling wind erosion susceptibility of Eastern Iran using machine learning
Source: PLoS One. 2026 Aug 6;21(8):e0354288. doi: 10.1371/journal.pone.0354288 (PMC13446583; doi:10.1371/journal.pone.0354288)
Supplement: Supplementary file 2 [file pone.0354288.s007.doc]

**Pointwise Reply to the comments made by the Reviewers:**

We would like to express our sincere gratitude to the Reviewers for their time and the constructive feedback provided. We have carefully addressed each of their comments and suggestions in the revised manuscript. We believe these revisions have significantly strengthened the paper and hope the reviewers find the updated version suitable for publication. We remain open to any further suggestions. The point wise response to the comments are mentioned in blue colour font.

**Lines 33-36. Authors may also search for and use more recent references for the dust impacts. Literature is rich in this field:**

**Response:** We agree with the reviewer that updating the socio-economic impacts of dust is vital. We have integrated the recent findings of *Yousefi et al. (2025)* and Mohammadi et al. (2025), which directly discuss multi-decadal aerosol changes and regional economic and health vulnerabilities.

**Lines 43-44. You should specify more about this statement. In which regions and periods? You should also support it with references:**

**Response:** We have specified the spatial and temporal context of the rising dust trends. The text now clearly highlights the Middle East and Southwest Asia over the last two decades, substantiated by Gholami et al. (2023) and Boroughani et al. (2025).

**Similarly, statements in lines 52-55 need more region-based citations:**

**Response:** Evaluated and corrected. We have reinforced the region-based characteristics of wind erosion inside Iran by invoking the threshold velocity and geomorphological baselines from Emamian et al. (2021) and Gholami et al. (2024b).

**Lines 80-81. I do not agree with this statement. There are numerous studies that assess the wind-erosion mapping in Iran. Some are cited in this study, for other authors may see the suggested literature:**

**Response:** We completely agree with the reviewer and apologize for the overstatement. We have rephrased this section to acknowledge the regional literature on Iranian dust mapping, while re-stating our study's unique value as a local-scale high-resolution comparative machine learning ensemble framework.

**Lines 96-100. Here, authors refer to previous works, their objectives and scientific gaps. However, they do not cite any of these previous works. In general, this paragraph, as well as the whole introduction section needs more comprehensive writing mostly on supporting the current writing and general statements with regional literature:**

**Response:** Concurred. To support our statements on the limitations of conventional approaches and the necessity of capturing complex, non-linear environmental drivers, we have integrated citations from Gholami et al. (2024a), which specifically introduced explainable artificial intelligence (XAI) to unravel these interactions.

**Line 129. This sentence is incomplete and needs revision:**

**Response:** We apologize for this typographical omission. The sentence has been completed.

**Lines 131-133. It's not clear if you refer to annual temperatures here. Also, authors refer to the statistical period, without mentioning it:**

**Response:** Clarified. We have updated the temperature descriptions to indicate they represent annual mean values and explicitly mentioned our long-term baseline period (2000–2020).

**Lines 135-146. Once again, these statements need to be supported by appropriate references in the region:**

**Response:** We have substantiated the environmental and geomorphological descriptions of Khorasan Province by referencing the local-scale sand/dust storm dynamics from Boroughani et al. (2025) and Mohammadi et al. (2025).

**Section 2.3. All these parameters described here are potential factors for wind erosion according to regional and global literature. So, for supporting all these implementing factors, also may cite some studies systematically used them for mapping wind erosion hazard in Iran and globally:**

**Response:** We have linked our theoretical parameter selection process to the advanced regional and global literature (Gholami et al., 2021; Boroughani et al., 2025), confirming that these edaphic, bioclimatic, and topographic variables are widely established as foundational drivers of aeolian hazards.

**Line 218. You should expand these abbreviations or include all variables in a nomenclature to be shown in the main text and not in supplementary material:**

**Response:** Thank you for highlighting this crucial detail. We have explicitly stated our grid harmonization and interpolation steps in Section 2.3 to clarify how variables with different pixel sizes were aligned.

**Section 2.4 is well-organized and structured; however, authors may support some statements or model groups with references, connecting their modelling approach with regional and global literature:**

**Response:** We have connected our tree-based, regression, and advanced non-parametric computational groupings with the state-of-the-art literature by citing Gholami & Mohammadifar (2022) and Yousefi et al. (2025).

**The beginning of the results section should be rather "smoothed", since the current writing does not refer to the study area, but generally, this confuses the reader to follow the following material. Authors may also refer to the objective of the study and the area of investigation:**

**Response:** We have modified the opening paragraph of the Results and Discussion section to immediately contextually bridge the text back to the specific territorial boundary of the Khorasan Province.

**Line 428. It's not clear what figure author refers to here. Definitely not to figure 3, it seems like something else, while till now they do not mention fig. 4. So, more detailed and comprehensive writing is needed here:**

**Response:** We apologize for the lack of clarity. The typographical errors in lines 441–446 have been checked and explicitly linked to the true graphics (Figure 3 for Feature Importance and Figure 4 for Spatial Patterns)**.**

**Line 547. Correct as "were consistent":**

**Response:** Done. The grammatical verb agreement has been corrected to "were consistent".

**Lines 548-550. You may revise this sentence by including references, as well as other studies from the recommended literature into a parenthesis like... Previous studies in Iran (...) also mentioned that ...:**

**Response:** Done. The regional citations confirming Random Forest efficiency have been properly enclosed in parentheses.

**Lines 580-592 constitute excellent interpretations and discussions about socio-economic impacts of land degradation in the east of Iran. Some of the sentences could be accompanied by relevant references to support them:**

**Response:** We appreciate the reviewer's praise for this socio-economic discussion. We have anchored these statements by citing Emamian et al. (2021) and Yousefi et al. (2025), who explicitly linked severe aridity and ecological resource depletion to regional job loss and rural-to-urban displacement.
