# Supplementary material for: Modeling wind erosion susceptibility of Eastern Iran using machine learning
Source: PLoS One. 2026 Aug 6;21(8):e0354288. doi: 10.1371/journal.pone.0354288 (PMC13446583; doi:10.1371/journal.pone.0354288)
Supplement: Peer review history

# Peer review: Decision Letter 0

**DOI:** 10.1371/journal.pone.0354288.r001

**Authors:** Judi Hewitt

17 May 2026

PONE-D-26-05438

Modeling Wind Erosion Susceptibility of Eastern Iran Using Machine Learning

PLOS One

Dear Dr. Shukla,

Thank you for submitting your manuscript to PLOS ONE. After careful consideration, we feel that it has merit but does not fully meet PLOS ONE’s publication criteria as it currently stands. Therefore, we invite you to submit a revised version of the manuscript that addresses the points raised during the review process.

There are several areas which the reviewer highlighted could be supported by references.  The major issue to be expanded upon was whether the different predictor variables differed in their spatial resolution, how this was dealt with and whether the particular learning methods are better able to deal with these

Please submit your revised manuscript by Jul 01 2026 11:59PM. If you will need more time than this to complete your revisions, please reply to this message or contact the journal office at plosone@plos.org. When you're ready to submit your revision, log on to https://www.editorialmanager.com/pone/ and select the 'Submissions Needing Revision' folder to locate your manuscript file.

Please include the following items when submitting your revised manuscript:

- A letter that responds to each point raised by the academic editor and reviewer(s). You should upload this letter as a separate file labeled 'Response to Reviewers'.
- A marked-up copy of your manuscript that highlights changes made to the original version. You should upload this as a separate file labeled 'Revised Manuscript with Track Changes'.
- An unmarked version of your revised paper without tracked changes. You should upload this as a separate file labeled 'Manuscript'.

If you would like to make changes to your financial disclosure, please include your updated statement in your cover letter. Guidelines for resubmitting your figure files are available below the reviewer comments at the end of this letter.

If applicable, we recommend that you deposit your laboratory protocols in protocols.io to enhance the reproducibility of your results. Protocols.io assigns your protocol its own identifier (DOI) so that it can be cited independently in the future. For instructions see: https://journals.plos.org/plosone/s/submission-guidelines#loc-laboratory-protocols. Additionally, PLOS ONE offers an option for publishing peer-reviewed Lab Protocol articles, which describe protocols hosted on protocols.io. Read more information on sharing protocols at https://plos.org/protocols?utm_medium=editorial-email&utm_source=authorletters&utm_campaign=protocols.

As the corresponding author, your ORCID iD is verified in the submission system and will appear in the published article. PLOS supports the use of ORCID, and we encourage all coauthors to register for an ORCID iD and use it as well. Please encourage your coauthors to verify their ORCID iD within the submission system before final acceptance, as unverified ORCID iDs will not appear in the published article. *Only*   the individual author can complete the verification step; PLOS staff *cannot*   verify ORCID iDs on behalf of authors.

We look forward to receiving your revised manuscript.

Kind regards,

Judi Hewitt, Hewitt

Academic Editor

PLOS One

**Journal Requirements:**

When submitting your revision, we need you to address these additional requirements.

1. Please ensure that your manuscript meets PLOS ONE's style requirements, including those for file naming. The PLOS ONE style templates can be found at

https://journals.plos.org/plosone/s/file?id=wjVg/PLOSOne_formatting_sample_main_body.pdf and

https://journals.plos.org/plosone/s/file?id=ba62/PLOSOne_formatting_sample_title_authors_affiliations.pdf

2. In your Methods section, please provide additional information regarding the permits you obtained for the work. Please ensure you have included the full name of the authority that approved the field site access and, if no permits were required, a brief statement explaining why.

3. Please include a complete copy of PLOS’ questionnaire on inclusivity in global research in your revised manuscript. Our policy for research in this area aims to improve transparency in the reporting of research performed outside of researchers’ own country or community. The policy applies to researchers who have travelled to a different country to conduct research, research with Indigenous populations or their lands, and research on cultural artefacts. The questionnaire can also be requested at the journal’s discretion for any other submissions, even if these conditions are not met.  Please find more information on the policy and a link to download a blank copy of the questionnaire here: https://journals.plos.org/plosone/s/best-practices-in-research-reporting. Please upload a completed version of your questionnaire as Supporting Information when you resubmit your manuscript.

4. Please note that PLOS One has specific guidelines on code sharing for submissions in which author-generated code underpins the findings in the manuscript. In these cases, we expect all author-generated code to be made available without restrictions upon publication of the work. Please review our guidelines at https://journals.plos.org/plosone/s/materials-and-software-sharing#loc-sharing-code and ensure that your code is shared in a way that follows best practice and facilitates reproducibility and reuse.

5. We note that your Data Availability Statement is currently as follows:

“All relevant data are within the manuscript and its Supporting Information files.”

Please confirm at this time whether or not your submission contains all raw data required to replicate the results of your study. Authors must share the “minimal data set” for their submission. PLOS defines the minimal data set to consist of the data required to replicate all study findings reported in the article, as well as related metadata and methods (https://journals.plos.org/plosone/s/data-availability#loc-minimal-data-set-definition).

For example, authors should submit the following data:

- The values behind the means, standard deviations and other measures reported;

- The values used to build graphs;

- The points extracted from images for analysis.

Authors do not need to submit their entire data set if only a portion of the data was used in the reported study.

If your submission does not contain these data, please either upload them as Supporting Information files or deposit them to a stable, public repository and provide us with the relevant URLs, DOIs, or accession numbers. For a list of recommended repositories, please see https://journals.plos.org/plosone/s/recommended-repositories.

If there are ethical or legal restrictions on sharing a de-identified data set, please explain them in detail (e.g., data contain potentially sensitive information, data are owned by a third-party organization, etc.) and who has imposed them (e.g., an ethics committee). Please also provide contact information for a data access committee, ethics committee, or other institutional body to which data requests may be sent. If data are owned by a third party, please indicate how others may request data access.

6. PLOS requires an ORCID iD for the corresponding author in Editorial Manager on papers submitted after December 6th, 2016. Please ensure that you have an ORCID iD and that it is validated in Editorial Manager. To do this, go to ‘Update my Information’ (in the upper left-hand corner of the main menu), and click on the Fetch/Validate link next to the ORCID field. This will take you to the ORCID site and allow you to create a new iD or authenticate a pre-existing iD in Editorial Manager.

7. Please amend your authorship list in your manuscript file to include the authors.

8. Please amend your list of authors on the manuscript to ensure that each author is linked to an affiliation. Authors’ affiliations should reflect the institution where the work was done (if authors moved subsequently, you can also list the new affiliation stating “current affiliation:….” as necessary).

9. We note that Figures 1 and 4 in your submission contain map images which may be copyrighted. All PLOS content is published under the Creative Commons Attribution License (CC BY 4.0), which means that the manuscript, images, and Supporting Information files will be freely available online, and any third party is permitted to access, download, copy, distribute, and use these materials in any way, even commercially, with proper attribution. For these reasons, we cannot publish previously copyrighted maps or satellite images created using proprietary data, such as Google software (Google Maps, Street View, and Earth). For more information, see our copyright guidelines: http://journals.plos.org/plosone/s/licenses-and-copyright.

We require you to either (1) present written permission from the copyright holder to publish these figures specifically under the CC BY 4.0 license, or (2) remove the figures from your submission:

1. You may seek permission from the original copyright holder of Figure(s) [#] to publish the content specifically under the CC BY 4.0 license.

We recommend that you contact the original copyright holder with the Content Permission Form (http://journals.plos.org/plosone/s/file?id=7c09/content-permission-form.pdf) and the following text:

“I request permission for the open-access journal PLOS ONE to publish XXX under the Creative Commons Attribution License (CCAL) CC BY 4.0 (http://creativecommons.org/licenses/by/4.0/). Please be aware that this license allows unrestricted use and distribution, even commercially, by third parties. Please reply and provide explicit written permission to publish XXX under a CC BY license and complete the attached form.”

Please upload the completed Content Permission Form or other proof of granted permissions as an "Other" file with your submission.

In the figure caption of the copyrighted figure, please include the following text: “Reprinted from [ref] under a CC BY license, with permission from [name of publisher], original copyright [original copyright year].”

2. If you are unable to obtain permission from the original copyright holder to publish these figures under the CC BY 4.0 license or if the copyright holder’s requirements are incompatible with the CC BY 4.0 license, please either i) remove the figure or ii) supply a replacement figure that complies with the CC BY 4.0 license. Please check copyright information on all replacement figures and update the figure caption with source information. If applicable, please specify in the figure caption text when a figure is similar but not identical to the original image and is therefore for illustrative purposes only.

The following resources for replacing copyrighted map figures may be helpful:

USGS National Map Viewer (public domain): http://viewer.nationalmap.gov/viewer/

The Gateway to Astronaut Photography of Earth (public domain): http://eol.jsc.nasa.gov/sseop/clickmap/

Maps at the CIA (public domain): https://www.cia.gov/library/publications/the-world-factbook/index.html and https://www.cia.gov/library/publications/cia-maps-publications/index.html

NASA Earth Observatory (public domain): http://earthobservatory.nasa.gov/

Landsat: http://landsat.visibleearth.nasa.gov/

USGS EROS (Earth Resources Observatory and Science (EROS) Center) (public domain): http://eros.usgs.gov/#

Natural Earth (public domain): http://www.naturalearthdata.com/

10. Please upload a copy of Figure 5, to which you refer in your text on page 13. If the figure is no longer to be included as part of the submission please remove all reference to it within the text.

11. Please upload a copy of Supporting Information Figures 1 and 2, Supplementary Table S1   which you refer to in your text on pages 8 and 15.

12. If the reviewer comments include a recommendation to cite specific previously published works, please review and evaluate these publications to determine whether they are relevant and should be cited. There is no requirement to cite these works unless the editor has indicated otherwise.

[Note: HTML markup is below. Please do not edit.]

Reviewers' comments:

Reviewer's Responses to Questions

**Comments to the Author**

1. Is the manuscript technically sound, and do the data support the conclusions?

The manuscript must describe a technically sound piece of scientific research with data that supports the conclusions. Experiments must have been conducted rigorously, with appropriate controls, replication, and sample sizes. The conclusions must be drawn appropriately based on the data presented.

Reviewer #1: Yes

**********

2. Has the statistical analysis been performed appropriately and rigorously?

Reviewer #1: Yes

**********

3. Have the authors made all data underlying the findings in their manuscript fully available?

The PLOS Data policy requires authors to make all data underlying the findings described in their manuscript fully available without restriction, with rare exception (please refer to the Data Availability Statement in the manuscript PDF file). The data should be provided as part of the manuscript or its supporting information, or deposited to a public repository. For example, in addition to summary statistics, the data points behind means, medians and variance measures should be available. If there are restrictions on publicly sharing data—e.g. participant privacy or use of data from a third party—those must be specified.

Reviewer #1: No

**********

4. Is the manuscript presented in an intelligible fashion and written in standard English?

PLOS ONE does not copyedit accepted manuscripts, so the language in submitted articles must be clear, correct, and unambiguous. Any typographical or grammatical errors should be corrected at revision, so please note any specific errors here.

Reviewer #1: Yes

**********

5. Review Comments to the Author

Please use the space provided to explain your answers to the questions above. You may also include additional comments for the author, including concerns about dual publication, research ethics, or publication ethics. (Please upload your review as an attachment if it exceeds 20,000 characters)

Reviewer #1: This study examined and maps the wind erosion susceptibility in the eastern part of Iran using 10 machine learning models, with more emphasis on Random Forest, which outperforms the others, and an ensemble of models. Authors also identify the areas with highest susceptibility to wind erosion and propose some restoration techniques and projects, that is especially important for this region. This is an interesting, well-organized and written work, that contributes to several previous works over Iran and the Middle East dealing with this issue. However, despite the rich literature over the region (see my recommendations below), the NE part of Iran was remained nearly unexplored, so the current paper has its own importance, which however, should be better stated and highlighted by the authors at the end of the Introduction section. I propose major revisions on this manuscript in a way to improve the scientific importance, connection with previous works and clarification of some statements and results.

Authors may also see results from similar studies in wind erosion hazard over Iranian provinces and specific regions of high land degradation and vulnerability to dust events and compare with current findings.

Boroughani et al. 2025. https://doi.org/10.1016/j.apr.2024.102380

Gholami et al. 2021. https://doi.org/10.1016/j.apr.2021.03.005

Gholami et al. 2023.doi: 10.3389/fenvs.2023.1187658

Gholami et al. 2024. https://doi.org/10.1016/j.aeolia.2024.100924

Gholami et al. 2024. https://doi.org/10.1038/s41598-024-70125-y

Emamian et al. 2021. https://doi.org/10.1016/j.ecolind.2020.107325

Yousefi et al. 2024. https://doi.org/10.1016/j.jenvman.2025.124346.

Mohammadi et al. 2025. https://doi.org/10.1016/j.aeolia.2025.100995

Lines 33-36. Authors may also search for and use more recent references for the dust impacts. Literature is rich in this field.

Lines 43-44. You should specify more about this statement. In which regions and periods? You should also support it with references.

Similarly, statements in lines 52-55 need more region-based citations.

Lines 80-81. I do not agree with this statement. There are numerous studies that assess the wind-erosion mapping in Iran. Some are cited in this study, for other authors may see the suggested literature.

Lines 96-100. Here, authors refer to previous works, their objectives and scientific gaps. However, they do not cite any of these previous works. In general, this paragraph, as well as the whole introduction section needs more comprehensive writing mostly on supporting the current writing and general statements with regional literature.

Line 129. This sentence is incomplete and needs revision.

Lines 131-133. It's not clear if you refer to annual temperatures here. Also, authors refer to the statistical period, without mentioning it.

Lines 135-146. Once again, these statements need to be supported by appropriate references in the region.

Section 2.3. All these parameters described here are potential factors for wind erosion according to regional and global literature. So, for supporting all these implementing factors, also may cite some studies systematically used them for mapping wind erosion hazard in Iran and globally.

Line 218. You should expand these abbreviations or include all variables in a nomenclature to be shown in the main text and not in supplementary material.

in Section 2.3, authors should also provide information about how do they overcome the issue of different spatial resolutions between the effective factors (variables) used in this analysis. What interpolation or extrapolation method did they use or any other technique for consistency of spatial resolution.

Section 2.4 is well-organized and structured; however, authors may support some statements or model groups with references, connecting their modelling approach with regional and global literature.

The beginning of the results section should be rather "smoothed", since the current writing does not refer to the study area, but generally, this confuses the reader to follow the following material. Authors may also refer to the objective of the study and the area of investigation.

Line 428. It's not clear what figure author refers to here. Definitely not to figure 3, it seems like something else, while till now they do not mention fig. 4. So, more detailed and comprehensive writing is needed here.

Line 547. Correct as "were consistent"

Lines 548-550. You may revise this sentence by including references, as well as other studies from the recommended literature into a parenthesis like... Previous studies in Iran (...) also mentioned that ...

Lines 580-592 constitute excellent interpretations and discussions about socio-economic impacts of land degradation in the east of Iran. Some of the sentences could be accompanied by relevant references to support them.

**********

6. PLOS authors have the option to publish the peer review history of their article (what does this mean?). If published, this will include your full peer review and any attached files.

If you choose “no”, your identity will remain anonymous but your review may still be made public.

**Do you want your identity to be public for this peer review?**   For information about this choice, including consent withdrawal, please see our Privacy Policy.

Reviewer #1: No

**********

[NOTE: If reviewer comments were submitted as an attachment file, they will be attached to this email and accessible via the submission site. Please log into your account, locate the manuscript record, and check for the action link "View Attachments". If this link does not appear, there are no attachment files.]

To ensure your figures meet our technical requirements, please review our figure guidelines: https://journals.plos.org/plosone/s/figures

You may also use PLOS’s free figure tool, NAAS, to help you prepare publication quality figures: https://journals.plos.org/plosone/s/figures#loc-tools-for-figure-preparation.

NAAS will assess whether your figures meet our technical requirements by comparing each figure against our figure specifications.

# Author comment: Author response to Decision Letter 1

**DOI:** 10.1371/journal.pone.0354288.r002

30 Jun 2026

Pointwise Reply to the comments made by the Reviewers:

We would like to express our sincere gratitude to the Reviewers for their time and the constructive feedback provided. We have carefully addressed each of their comments and suggestions in the revised manuscript. We believe these revisions have significantly strengthened the paper and hope the reviewers find the updated version suitable for publication. We remain open to any further suggestions. The point wise response to the comments are mentioned in blue colour font.

Lines 33-36. Authors may also search for and use more recent references for the dust impacts. Literature is rich in this field:

Response: We agree with the reviewer that updating the socio-economic impacts of dust is vital. We have integrated the recent findings of Yousefi et al. (2025) and Mohammadi et al. (2025), which directly discuss multi-decadal aerosol changes and regional economic and health vulnerabilities.

Lines 43-44. You should specify more about this statement. In which regions and periods? You should also support it with references:

Response: We have specified the spatial and temporal context of the rising dust trends. The text now clearly highlights the Middle East and Southwest Asia over the last two decades, substantiated by Gholami et al. (2023) and Boroughani et al. (2025).

Similarly, statements in lines 52-55 need more region-based citations:

Response: Evaluated and corrected. We have reinforced the region-based characteristics of wind erosion inside Iran by invoking the threshold velocity and geomorphological baselines from Emamian et al. (2021) and Gholami et al. (2024b).

Lines 80-81. I do not agree with this statement. There are numerous studies that assess the wind-erosion mapping in Iran. Some are cited in this study, for other authors may see the suggested literature:

Response: We completely agree with the reviewer and apologize for the overstatement. We have rephrased this section to acknowledge the regional literature on Iranian dust mapping, while re-stating our study's unique value as a local-scale high-resolution comparative machine learning ensemble framework.

Lines 96-100. Here, authors refer to previous works, their objectives and scientific gaps. However, they do not cite any of these previous works. In general, this paragraph, as well as the whole introduction section needs more comprehensive writing mostly on supporting the current writing and general statements with regional literature:

Response: Concurred. To support our statements on the limitations of conventional approaches and the necessity of capturing complex, non-linear environmental drivers, we have integrated citations from Gholami et al. (2024a), which specifically introduced explainable artificial intelligence (XAI) to unravel these interactions.

Line 129. This sentence is incomplete and needs revision:

Response: We apologize for this typographical omission. The sentence has been completed.

Lines 131-133. It's not clear if you refer to annual temperatures here. Also, authors refer to the statistical period, without mentioning it:

Response: Clarified. We have updated the temperature descriptions to indicate they represent annual mean values and explicitly mentioned our long-term baseline period (2000–2020).

Lines 135-146. Once again, these statements need to be supported by appropriate references in the region:

Response: We have substantiated the environmental and geomorphological descriptions of Khorasan Province by referencing the local-scale sand/dust storm dynamics from Boroughani et al. (2025) and Mohammadi et al. (2025).

Section 2.3. All these parameters described here are potential factors for wind erosion according to regional and global literature. So, for supporting all these implementing factors, also may cite some studies systematically used them for mapping wind erosion hazard in Iran and globally:

Response: We have linked our theoretical parameter selection process to the advanced regional and global literature (Gholami et al., 2021; Boroughani et al., 2025), confirming that these edaphic, bioclimatic, and topographic variables are widely established as foundational drivers of aeolian hazards.

Line 218. You should expand these abbreviations or include all variables in a nomenclature to be shown in the main text and not in supplementary material:

Response: Thank you for highlighting this crucial detail. We have explicitly stated our grid harmonization and interpolation steps in Section 2.3 to clarify how variables with different pixel sizes were aligned.

Section 2.4 is well-organized and structured; however, authors may support some statements or model groups with references, connecting their modelling approach with regional and global literature:

Response: We have connected our tree-based, regression, and advanced non-parametric computational groupings with the state-of-the-art literature by citing Gholami & Mohammadifar (2022) and Yousefi et al. (2025).

The beginning of the results section should be rather "smoothed", since the current writing does not refer to the study area, but generally, this confuses the reader to follow the following material. Authors may also refer to the objective of the study and the area of investigation:

Response: We have modified the opening paragraph of the Results and Discussion section to immediately contextually bridge the text back to the specific territorial boundary of the Khorasan Province.

Line 428. It's not clear what figure author refers to here. Definitely not to figure 3, it seems like something else, while till now they do not mention fig. 4. So, more detailed and comprehensive writing is needed here:

Response: We apologize for the lack of clarity. The typographical errors in lines 441–446 have been checked and explicitly linked to the true graphics (Figure 3 for Feature Importance and Figure 4 for Spatial Patterns).

Line 547. Correct as "were consistent":

Response: Done. The grammatical verb agreement has been corrected to "were consistent".

Lines 548-550. You may revise this sentence by including references, as well as other studies from the recommended literature into a parenthesis like... Previous studies in Iran (...) also mentioned that ...:

Response: Done. The regional citations confirming Random Forest efficiency have been properly enclosed in parentheses.

Lines 580-592 constitute excellent interpretations and discussions about socio-economic impacts of land degradation in the east of Iran. Some of the sentences could be accompanied by relevant references to support them:

Response: We appreciate the reviewer's praise for this socio-economic discussion. We have anchored these statements by citing Emamian et al. (2021) and Yousefi et al. (2025), who explicitly linked severe aridity and ecological resource depletion to regional job loss and rural-to-urban displacement.

Attachment Submitted filename: Response to Reviewers.doc

# Peer review: Decision Letter 1

**DOI:** 10.1371/journal.pone.0354288.r003

**Authors:** Judi Hewitt

7 Jul 2026

Modeling Wind Erosion Susceptibility of Eastern Iran Using Machine Learning

PONE-D-26-05438R1

Dear Dr. Shukla,

We’re pleased to inform you that your manuscript has been judged scientifically suitable for publication and will be formally accepted for publication once it meets all outstanding technical requirements.

Within one week, you’ll receive an e-mail detailing the required amendments. When these have been addressed, you’ll receive a formal acceptance letter and your manuscript will be scheduled for publication.

An invoice will be generated when your article is formally accepted. Please note, if your institution has a publishing partnership with PLOS and your article meets the relevant criteria, all or part of your publication costs will be covered. Please make sure your user information is up-to-date by logging into Editorial Manager at Editorial Manager® and clicking the ‘Update My Information' link at the top of the page. For questions related to billing, please contact billing support.

If your institution or institutions have a press office, please notify them about your upcoming paper to help maximize its impact. If they’ll be preparing press materials, please inform our press team as soon as possible -- no later than 48 hours after receiving the formal acceptance. Your manuscript will remain under strict press embargo until 2 pm Eastern Time on the date of publication. For more information, please contact onepress@plos.org.

Kind regards,

Judi Hewitt, Hewitt

Academic Editor

PLOS One

Additional Editor Comments (optional):

Reviewers' comments:

Reviewer's Responses to Questions

**Comments to the Author**

1. If the authors have adequately addressed your comments raised in a previous round of review and you feel that this manuscript is now acceptable for publication, you may indicate that here to bypass the “Comments to the Author” section, enter your conflict of interest statement in the “Confidential to Editor” section, and submit your "Accept" recommendation.

Reviewer #1: All comments have been addressed

**********

2. Is the manuscript technically sound, and do the data support the conclusions?

The manuscript must describe a technically sound piece of scientific research with data that supports the conclusions. Experiments must have been conducted rigorously, with appropriate controls, replication, and sample sizes. The conclusions must be drawn appropriately based on the data presented.

Reviewer #1: Yes

**********

3. Has the statistical analysis been performed appropriately and rigorously?

Reviewer #1: Yes

**********

4. Have the authors made all data underlying the findings in their manuscript fully available?

The PLOS Data policy requires authors to make all data underlying the findings described in their manuscript fully available without restriction, with rare exception (please refer to the Data Availability Statement in the manuscript PDF file). The data should be provided as part of the manuscript or its supporting information, or deposited to a public repository. For example, in addition to summary statistics, the data points behind means, medians and variance measures should be available. If there are restrictions on publicly sharing data—e.g. participant privacy or use of data from a third party—those must be specified.

Reviewer #1: Yes

**********

5. Is the manuscript presented in an intelligible fashion and written in standard English?

PLOS ONE does not copyedit accepted manuscripts, so the language in submitted articles must be clear, correct, and unambiguous. Any typographical or grammatical errors should be corrected at revision, so please note any specific errors here.

Reviewer #1: Yes

**********

6. Review Comments to the Author

Please use the space provided to explain your answers to the questions above. You may also include additional comments for the author, including concerns about dual publication, research ethics, or publication ethics. (Please upload your review as an attachment if it exceeds 20,000 characters)

Reviewer #1: (No Response)

**********

7. PLOS authors have the option to publish the peer review history of their article (what does this mean?). If published, this will include your full peer review and any attached files.

If you choose “no”, your identity will remain anonymous but your review may still be made public.

**Do you want your identity to be public for this peer review?**   For information about this choice, including consent withdrawal, please see our Privacy Policy.

Reviewer #1: No

**********

# Editor report: Acceptance letter

**DOI:** 10.1371/journal.pone.0354288.r004

**Authors:** Judi Hewitt

PONE-D-26-05438R1

PLOS One

Dear Dr. Shukla,

I'm pleased to inform you that your manuscript has been deemed suitable for publication in PLOS One. Congratulations! Your manuscript is now being handed over to our production team.

At this stage, our production department will prepare your paper for publication. This includes ensuring the following:

* All references, tables, and figures are properly cited

* All relevant supporting information is included in the manuscript submission,

* There are no issues that prevent the paper from being properly typeset

You will receive further instructions from the production team, including instructions on how to review your proof when it is ready. Please keep in mind that we are working through a large volume of accepted articles, so please give us a few days to review your paper and let you know the next and final steps.

Lastly, if your institution or institutions have a press office, please let them know about your upcoming paper now to help maximize its impact. If they'll be preparing press materials, please inform our press team within the next 48 hours. Your manuscript will remain under strict press embargo until 2 pm Eastern Time on the date of publication. For more information, please contact onepress@plos.org.

You will receive an invoice from PLOS for your publication fee after your manuscript has reached the completed accept phase. If you receive an email requesting payment before acceptance or for any other service, this may be a phishing scheme. Learn how to identify phishing emails and protect your accounts at https://explore.plos.org/phishing.

If we can help with anything else, please email us at customercare@plos.org.

Thank you for submitting your work to PLOS ONE and supporting open access.

Kind regards,

PLOS ONE Editorial Office Staff

on behalf of

Dr. Judi Hewitt, Hewitt

Academic Editor

PLOS One
