# Supplementary material for: Correction: Modeling wind erosion susceptibility of Eastern Iran using machine learning
Source: PLoS One. 2026 Oct 6;21(10):e0360012. doi: 10.1371/journal.pone.0360012 (PMC13641335; doi:10.1371/journal.pone.0360012)
Supplement: Supplementary file 1 [file pone.0360012.s001.pdf]

RESEARCH ARTICLE

Modeling wind erosion susceptibility of Eastern Iran using machine learning

Momeni Damaneh Javad[1☯], Tajbakhsh Fakhrabadi Seyed Mohammad[2☯], Memarian Hadi[2☯], Komeh Zinatplease[2☯], Dericks Praise Shukla[3,4*]

1 Department of Natural Resources Engineering, Faculty of Agriculture and Natural Resources, University of Hormozgan, Bandar Abbas, Iran, **2** Department of Rangeland and Watershed Management, Faculty of Natural Resources and Environment, University of Birjand, Birjand, Iran, **3** School of Civil and Environmental Engineering, IIT Mandi, Mandi, India, **4** Department of Civil Engineering, Pokhra Engineering College, Pokhra, Nepal

☯ These authors contributed equally to this work.
* dericks.shukla@pec.edu.np, dericks@iitmandi.ac.in

Abstract

Wind erosion and dust storms pose significant environmental and socio-economic threats to Iran's drylands, driven by both natural processes and human activities. This study addresses a critical gap by employing advanced machine learning (ML) techniques to generate high-resolution spatial susceptibility maps for wind erosion in the highly vulnerable eastern and northeastern regions of Iran. We evaluated ten distinct ML models using a comprehensive set of environmental predictors including climatic, soil, topographic, and geological variables to identify areas most prone to erosion. Our results demonstrated that the Random Forest (RF) and Ensemble (ESMs) models outperformed others, achieving high predictive accuracy based on ROC, TSS, and Kappa metrics. These models revealed that approximately 27–30% of the study area is susceptible to moderate or high-intensity wind erosion. Key drivers influencing erosion distribution were identified as soil texture (particularly sand content), specific bio-climatic factors (temperature seasonality and precipitation patterns), and topographic features such as elevation and wetness index. The resulting susceptibility maps provide a vital decision-support tool for policymakers and land managers, enabling targeted mitigation strategies in priority zones. Without effective intervention, these areas face accelerated land degradation, threatening agricultural sustainability and potentially exacerbating socio-economic challenges such as rural-to-urban migration. This study underscores the value of machine learning in environmental hazard assessment and offers a scalable framework for wind erosion management in arid regions worldwide.

Check for updates

OPEN ACCESS

Citation: Damaneh Javad M, Fakhrabadi Seyed Mohammad T, Hadi M, Zinatplease K, Shukla DP (2026) Modeling wind erosion susceptibility of Eastern Iran using machine learning. PLoS One 21(8): e0354288. https://doi.org/10.1371/journal.pone.0354288

Editor: Judi Hewitt, Hewitt, The University of Auckland - City Campus: University of Auckland, NEW ZEALAND

Received: January 31, 2026

Accepted: July 6, 2026

Published: August 6, 2026

Peer Review History: PLOS recognizes the benefits of transparency in the peer review process; therefore, we enable the publication of all of the content of peer review and author responses alongside final, published articles. The editorial history of this article is available here: https://doi.org/10.1371/journal.pone.0354288

Copyright: © 2026 Damaneh Javad et al. This is an open access article distributed under the terms of the Creative Commons Attribution License, which permits unrestricted use,

distribution, and reproduction in any medium, provided the original author and source are credited.

Data availability statement: All relevant data are within the manuscript and its Supporting information files.

Funding: The author(s) received no specific funding for this work.

Competing interests: The authors have declared that no competing interests exist.

Abbreviations: ANN, Artificial neural network; ASTER, Advanced Spaceborne Thermal Emission and Reflection Radiometer; BIO1–BIO19, Bioclimatic variables (WorldClim); CTA, Classification tree analysis; DEM, Digital elevation model; ESMs, Ensemble modeling technique; FDA, Flexible denotative analysis; GBM, Generalized boosting method; GIS, Geographic information systems; GLM, Generalized liner model; GPS, Global positioning system; H1, high intensity areas; H2, areas with medium intensity; H3, Low intensity; KAPPA, Kappa cohen; LST, Land surface temperature; MARS, Multivariate adaptive regression spline; MaxEnt, Maximum entropy model; NDVI, Normalized difference vegetation index; RF, Random forest; ROC, Receiver operating characteristic; SRE, Surface range envelope; TRI, Terrain ruggedness index; TSS, True skill statistic; TWI, Topographic wetness index.

Introduction

Dust storms primarily originate from arid and semi-arid regions, that are collectively called drylands, are integral components of the Earth system [1,2]. Drylands, which cover approximately 40% of the global terrestrial surface, are inhabited by over 2 billion people [3]. Due to the long-range transport of dust particles, which can travel thousands of kilometers, the environmental and socio-economic impact of dust storms extends far beyond the drylands themselves. These storms influence a variety of Earth system processes, such as climate dynamics, nutrient cycling, biogeochemical interactions, soil properties, and geomorphology, all of which have a significant effect on human health, urban infrastructures, and regional economies [4–6]. In recent decades, the scientific understanding of desert dust has expanded significantly due to its effects on human health, ecosystems, and climate [7]. These dust emissions are influenced by seasonal cycles, climatic events such as droughts [8], and large-scale atmospheric oscillations, including the North Atlantic Oscillation [9]. Dust storms generated by wind erosion affect terrestrial and marine ecosystems, as well as weather phenomena and climate dynamics [10–12]. Recent trends indicate a critical increase in the frequency and intensity of dust storms over the last two decades, particularly across hyper-arid zones in Southwest Asia and the Middle East, prompting international attention [13,14]. The United Nations General Assembly (UNGA) recognized the growing importance of dust storms in 2015 with the adoption of Resolution A/RES/70/195, titled “Combating Sand and Dust Storms”. This was followed by further action during the second UN Environment Assembly (UNEA-2) in 2016 [15], which called on member states to address the challenges posed by dust storms through policy initiatives [10,11].

Dust storms are frequent in regions such as the Sahara Desert, Central and Eastern Asia, the Middle East, and parts of the western United States, showing severe geomorphological and aerodynamic variability across eastern Iranian provinces [16,17]. They affect major urban centers in downwind regions, cities such as Phoenix, Athens, Dubai, Tehran, Beijing, and Sydney are routinely affected by dust storms, which can carry particulate matter, pollutants, and allergens over vast distances, leading to air quality issues and posing significant health risks [18]. Moreover, land use changes and the effects of climate change have been shown to contribute to the growing frequency of dust storms in certain regions [19,20].

Iran is particularly vulnerable to dust storms, especially in its eastern and southeastern regions, which are subject to persistent wind erosion. Several factors contribute to the occurrence of dust storms in Iran. Natural factors such as topographic diversity, climatic variability, and several seasonal effective air masses have created a highly complex situation in this region [21]. The country’s predominantly arid and semi-arid climate, characterized by low annual precipitation often below 200 mm creates a conducive environment for wind erosion. In addition, global climate change and the increasing frequency of droughts have intensified the spatial temporal frequency of Sand and dust storms (SDS) events, particularly in the past few decades [19–22]. Human-induced factors, such as improper land management and unsustainable agricultural practices, have further exacerbated the intensity of dust storms in Iran [23].

Recent advancements in remote sensing, Geographic Information Systems (GIS), and machine learning algorithms have provided powerful tools for understanding and mapping dust sources in drylands. Studies have employed a variety of methodologies, such as statistical models, the Analytical Hierarchy Process (AHP), and logistic regression, to assess the susceptibility of regions to wind erosion and dust storm events [24,25]. Furthermore, recent advances in machine learning have enhanced the analysis for environmental modeling. For instance, studies in similar arid regions, such as those in the Sahara Desert [26] and Central Asia [27], have demonstrated the efficacy of ensemble methods and Random Forest in predicting dust emission zones.

While numerous valuable studies have successfully mapped wind erosion and dust susceptibility across various provincial scales in Iran [16,28], generating a unified high-resolution, multi-algorithmic comparative framework remains highly essential for prioritizing specific local mitigation efforts. Accurate assessments of wind erosion susceptibility are essential for prioritizing mitigation efforts, especially given the limitations of Iran's soil conservation budget and resources [29]. The absence of such data impedes effective policy making and could lead to the mis-allocation of resources for dust storm mitigation. For example, Cao et al. (2015) utilized indicators like the Normalized Difference Vegetation Index (NDVI) and Net Primary Productivity (NPP) to identify potential dust sources across Iran, with a focus on the central, southern, and southeastern regions, which are highly prone to dust storm events [30]. Additionally, recent studies by Jafari et al. (2022) and Boroughani et al. (2023) have used remote sensing data and machine learning algorithms to map areas at risk of soil erosion and land degradation, further demonstrating the utility of these approaches in predicting dust storm occurrences and guiding mitigation strategies [31,32].

Expanding upon these regional assessments, recent global and national literature underscores that Sand and Dust Storms (SDS) and wind erosion remain critically tied to accelerated land degradation and anthropogenic land use/land cover (LULC) alterations [5,13]. To mitigate these destructive impacts, the scientific paradigm has rapidly shifted from conventional multi-index remote sensing frameworks toward highly sophisticated computational methods. Early investigations successfully leveraged MODIS derived observations and regional climate models (RegCM4) to delineate hyper-sensitive SDS hotspots and evaluate foundational geomorphological determinants [13,28]. Building upon these physical and statistical baselines, contemporary research increasingly integrates deep learning architecture with graph convolutional networks (GCNs) and active learning techniques (RNN-GRU) to map spatial complexities and unveil previously overlooked risk zones globally [14,17]. More importantly, the current state-of-the-art incorporates explainable artificial intelligence (XAI), utilizing SHAP and Permutation Feature Importance, alongside multi decadal aerosol datasets (MERRA-2) and novel vegetation metrics like the Future Restoration Dispersal Index (FRDI) [6,16,17]. This methodological evolution not only provides interpretable insights into multi-factorial environmental drivers but also establishes robust, predictive frameworks essential for targeted land conservation and sustainable policy planning.

Despite the recognized severity of wind erosion in Iran and the growing body of research on the topic, a critical and persistent knowledge gap remains: the precise, high-resolution spatial identification of dust emission hotspots at a local scale, particularly in the highly vulnerable eastern and Northeastern regions. Although previous studies have broadly delineated susceptible zones, many have historically relied on conventional linear methodologies or a constrained suite of macro-environmental variables [24]. Such approaches often fail to capture the complex, non-linear interactions between the diverse climatic, topographic, pedological, and anthropogenic drivers that shape Iran's heterogeneous landscapes, a computational gap recently addressed globally through explainable frameworks [17]. Consequently, there is a pressing need for an advanced predictive modeling framework capable of not only generating accurate susceptibility maps but also quantitatively deciphering the relative contribution of each causative factor. To address this gap, this study aims to develop a high-resolution wind erosion susceptibility map for eastern and Northeastern Iran by employing a suite of machine learning algorithms including Maximum Entropy (MaxEnt), Generalized Linear Model (GLM), Random Forest (RF), Generalized Boosting Method (GBM), Multivariate Adaptive Regression Splines (MARS), Artificial Neural Networks (ANN), Flexible Discriminant Analysis (FDA), Classification Tree Analysis (CTA), Surface Range Envelope (SRE), and Generalized Additive

Model (GAM) to integrate a comprehensive multi-factorial datasets. This research is guided by two primary questions: 1) Which specific areas are at the highest risk of wind erosion? and 2) Which environmental parameters are the most influential predictors of erosion susceptibility? The findings are anticipated to provide a robust scientific foundation for effective and cost-efficient conservation strategies, enabling policymakers to prioritize interventions in the most critical areas. These maps are intended to inform targeted mitigation efforts and enhance the effectiveness of policies aimed at reducing the frequency and severity of dust storms in these vulnerable regions.

Materials and methods

Study area

The present work is conducted in the Khorasan Province that is situated in the eastern and northeastern geographical region of Iran, covering an area of 295,115 square kilometers. The study area is situated between 30° 31′54″ to 38°11′53″ N latitude and 55°26′36″ to 61°26′34″ E longitude (Fig 1). It is located in the Iranian-Turanian phytogeographical region and due to its vast expanse, exhibits a wide range of diverse natural conditions. Each of its different regions possesses specific ecological characteristics. According to climatic classification, the whole province is characterized by a cold desert climate, while some parts also exhibit a cold semi-desert climate which is a favourable condition for dust storms and wind erosion [33]. Generally, the climate of Khorasan Province is dry and cold, with an average annual precipitation of 210 millimeters recorded over the evaluated long-term statistical period. Precipitation distribution is not uniform throughout the province, with the overall amount decreasing from north to south. In terms of temperature, the minimum annual mean temperature of the province is 2.12°C, the maximum annual mean is 18.2°C during the baseline statistical period (2000–2020), and the overall annual mean temperature is 15.6°C [34].

This Province represents a critically vulnerable and highly representative region for wind erosion studies in Iran. Rangeland is the dominant land use in the region, with low-lying plains primarily composed of loose Quaternary alluvial and subterranean deposits that are highly prone to physical degradation and particle entrainment under anthropogenic LULC alterations [5,13]. Recent droughts have disrupted the hydrological cycle, reducing both surface water and groundwater availability [22]. Moreover, mismanagement of land and water resources through dam construction, diversion channels, and land use changes has elevated the situation, creating favorable conditions for dust storm formation [19,35]. Therefore, investigating the driving factors here is not only critical for local land management but also provides a model for understanding wind erosion processes in comparable vulnerable ecosystems throughout Iran.

Identification of wind erosion centers' locations

Field survey was carried out during 2018–2020 to collect the data of the locations that were prone to erosional activity. Those areas having predominant wind erosion and covered an area of at least 10,000 sq m were considered to be location of occurrences. These points were divided into areas of 3 intensities of wind erosion namely high intensity (H1), medium intensity (H2) and low intensity (H3). A total of 2,324 points were recorded for high-activity erosion areas (H1), 3,784 points for medium-activity erosion areas (H2), and 2,624 points for low-activity erosion areas (H3) using the Global Positioning System (GPS) (Fig 1). Then spatial filtering was applied to the identified erosion points to mitigate the potential effects of spatial autocorrelation and reduce sampling bias. The points were overlaid on a 2000 × 2000 m grid across the study area in ArcGIS 10.8. Only one presence point was retained per grid cell, ensuring a more uniform spatial distribution of the input data for the subsequent modeling phase [36]. No specific permits were required for the described field studies as the activities did not involve endangered species or protected private lands.

The flowchart, which explains the methodology used in this work is shown in Fig 2. Based on studies conducted by the Natural Resources and Watershed Management Organization of Iran in 2019, the characteristics of each geomorphological unit, including land type, vegetation cover, soil texture, presence of pebbles or crust, and other environmental factors

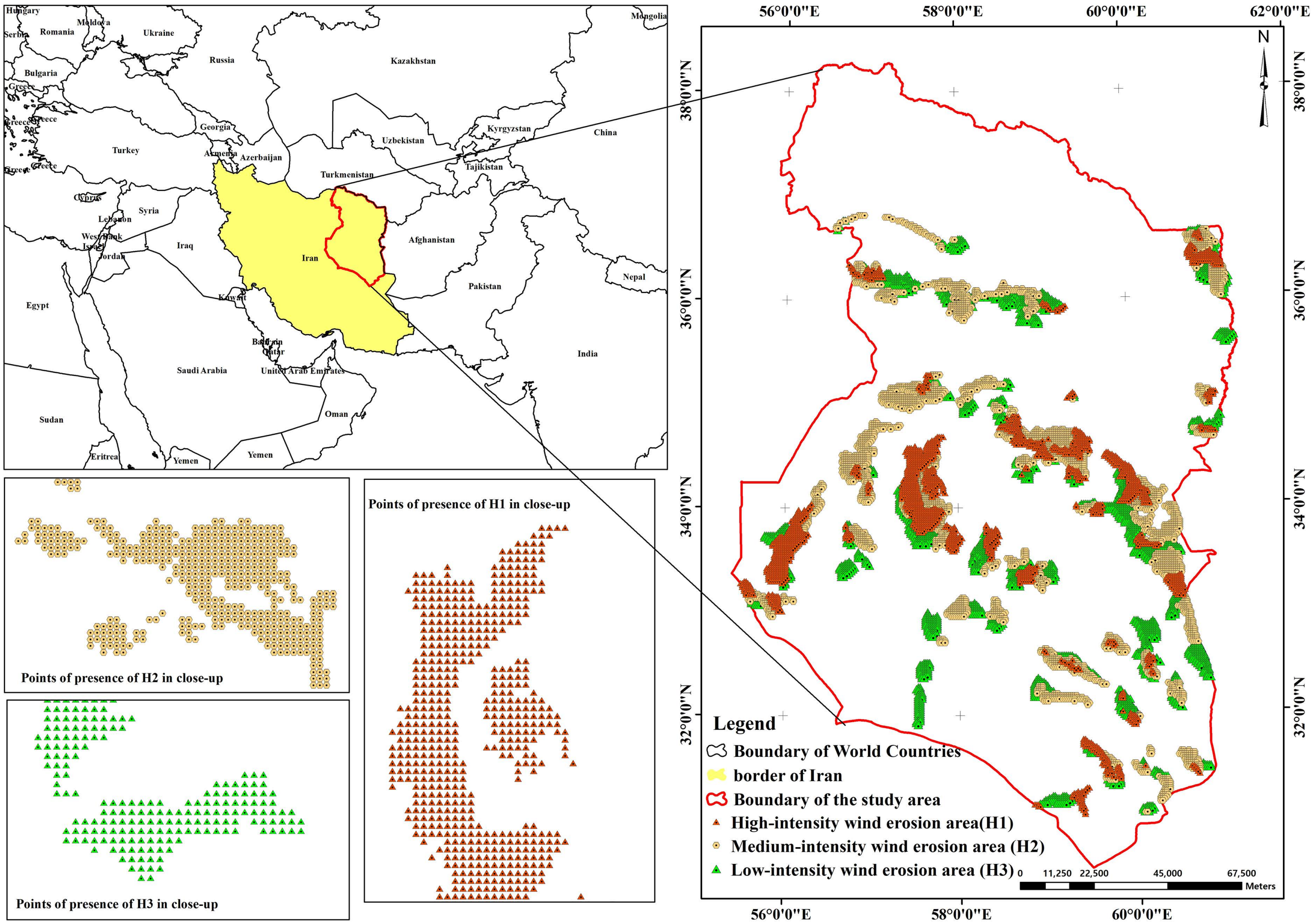

Fig 1. Geographic location of the study area and spatial distribution of wind erosion centers across eastern and northeastern Iran. (All spatial maps are original, generated by the authors, and formatted under CC BY 4.0 open-access public domain).

https://doi.org/10.1371/journal.pone.0354288.g001

that influence wind erosion, were determined. Subsequently, a quantitative score is assigned to each of the defined indicators. Based on the sum of the indicator scores in each geomorphological unit, the severity of wind erosion is determined and classified into three categories: low H3, medium H2 and high H1 (Provided by Iran's Natural Resources and Watershed Management Organization, 2019). Then, the total indicators of wind erosion detection were calculated using Pearson correlation for three classes of wind erosion: low (H3), medium (H2), and high (H1) (Supplementary S1, S2, S3 Figs). Finally, sensitivity maps were created using the selected environmental predictors and 10 different machine learning algorithms. The methodology and dataset are detailed in the following section.

Environmental variables determination

The identification of predictive variables for modeling wind erosion potential was conducted through a structured, two-phase process to ensure both scientific comprehensiveness and statistical robustness. An initial set of 90 environmental variables were selected based on an extensive review of established literature on aeolian processes and dust emission

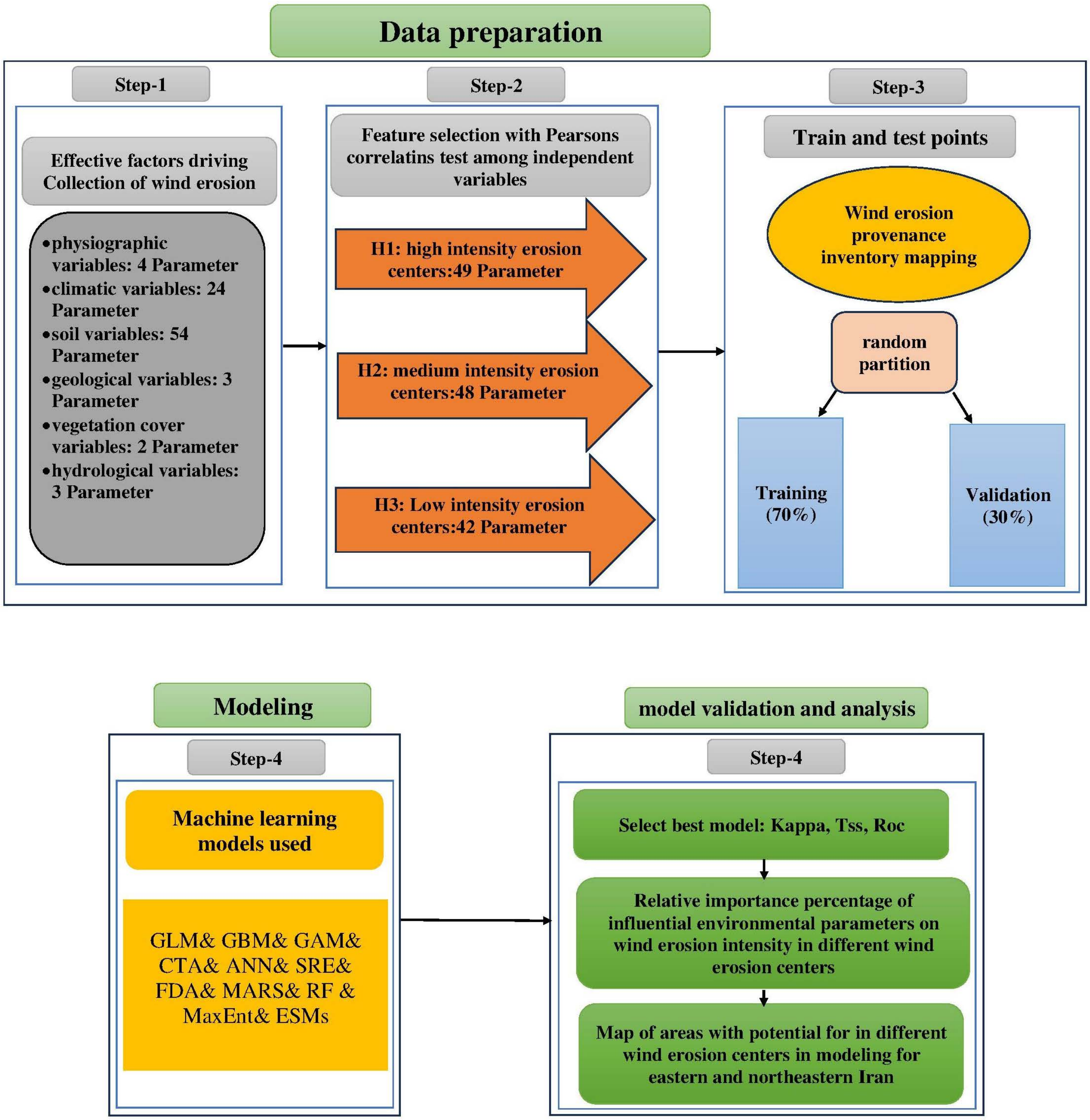

Fig 2. Flowchart of inputs, data processing and outputs in the present study.

https://doi.org/10.1371/journal.pone.0354288.g002

dynamics, alongside expert knowledge of the study area's specific biophysical conditions. These multi-factorial criteria are systematically corroborated by advanced national and global frameworks evaluating geomorphological and climatic erosion constraints [13,28]. This theory-driven selection aimed to capture the primary factors controlling wind erosion, including: climatic variables; soil; physiographic; geologic, vegetation and hydrological variables. Climatic variables include 24 factors such as temperature seasonality (BIO4) and precipitation of the driest quarter (BIO17), which directly influence

soil moisture and vegetation cover that are key determinants of soil erodibility. Soil variables (54 factors): include texture (sand, silt, clay content), organic carbon, and chemical properties (pH, cation exchange capacity), which define the soil's inherent susceptibility to deflation. Physiographic (4 factors) and Topographic variables: such as elevation, slope, and the Topographic Wetness Index (TWI), which affect wind flow patterns, sediment transport, and surface water retention. Geological (3 factors), Vegetation (2 factors), and Hydrological variables (3 factors) account for parent material, surface roughness from vegetation, and moisture availability, all of which modify erosion thresholds.

Data layers for these variables were taken from globally recognized, publicly available databases to ensure reproducibility. The nineteen bioclimatic variables (BIO1 to BIO19) were obtained from the WorldClim database (version 2.1) at a spatial resolution of 1 km² (www.worldclim.org). Wind speed data at various altitudes were retrieved from the Global Solar Atlas (2020; https://globalsolaratlas.info). Soil properties including physical characteristics such as bulk density, texture (sand, silt, clay), and coarse fragment content, as well as chemical attributes including nitrogen, organic carbon, pH, cation exchange capacity, and World Reference Base (WRB) soil classification were acquired from the SoilGrids database at 250 m resolution (https://soilgrids.org). Remotely sensed indices, such as NDVI, NDBI, NDMI, NDWI, and multiple salinity indices, along with Land Surface Temperature (LST), were processed using the Google Earth Engine platform based on Landsat 8 imagery. Geological data, including information on epoch, age, and formation type, were obtained from the Iran Geological Organization. Topographic parameters Altitude, Topographic Wetness Index (TWI), Terrain Ruggedness Index (TRI), and Slope were derived from the Advanced Spaceborne Thermal Emission and Reflection Radiometer (ASTER) Global Digital Elevation Model (GDEM) at 30 m resolution, downloaded via USGS Earth Explorer (https://earthexplorer.usgs.gov). A complete list of all variables, including their descriptions, abbreviations, units, and original data sources, is provided in Supplementary S1 Table.

All these data layers were harmonized to a consistent spatial resolution of 1000x1000 meters using Idrisi Selva software. To overcome the issue of varying spatial configurations, high-resolution predictors (e.g., 30 m ASTER GDEM) were upscaled via the Nearest Neighbor resampling method to match the target 1 km grid, thereby ensuring spatial alignment and consistency across all layers while accurately preserving original boundary pixel values without introducing artificial smoothing artifacts. To mitigate the adverse effects of multicollinearity and model over-fitting, a Pearson correlation analysis with a threshold of $|r| < 0.8$ was applied [34,37,38]. This objective statistical filtering resulted in a refined and robust subset of predictors for each erosion intensity class: 49 variables for high-intensity Regions (H1), 48 for medium-intensity (H2), and 42 for low-intensity (H3) (see S1 Fig for the workflow). This rigorous process ensures the final models are both ecologically relevant and statistically correct.

Modeling the distribution of wind erosion susceptible areas

The spatial distribution of areas susceptible to wind erosion with varying intensities (high, medium, and low) was modeled using 10 machine learning algorithms available in the BioMod2 software package [39]. To generate non-presence points, the BioMod software was employed. For model development, 70% of the presence points were utilized, while the remaining 30% were used for model evaluation. This procedure was repeated five times to improve model accuracy and minimize potential bias. The models were evaluated and compared based on key metrics such as ROC, TSS, and Kappa scores to identify the most effective algorithm for predicting wind erosion susceptibility [40]. The strategic selection of ten distinct machine learning algorithms was guided by a deliberate methodology aimed at capturing the complex and frequently non-linear nature of wind erosion processes through a diverse suite of computational approaches. This multi-model framework was implemented to ensure analytical robustness and to account for the inherent uncertainties present in ecological modeling. Initially, regression-based models, including Generalized Linear Models (GLM), Generalized Additive Models (GAM), and Multivariate Adaptive Regression Splines (MARS), were used to identify and model both linear and non-linear relationships between environmental predictors and erosion susceptibility, thereby providing a foundation of interpretable results. To further address complex variable interactions and intricate non-linear

patterns without requiring stringent prior assumptions about data distribution, tree-based ensemble models Random Forest (RF), Gradient Boosting Machine (GBM), and Classification Tree Analysis (CTA) were utilized for their proven power in predicting spatial phenomena. Complementing these, a group of advanced machine learning and non-parametric techniques, comprising Maximum Entropy (MaxEnt), Artificial Neural Networks (ANN), Flexible Discriminant Analysis (FDA), and Surface Range Envelope (SRE), was applied for their superior capacity to learn complex patterns directly from the data; MaxEnt is particularly suited for presence-only data, ANN can model highly intricate relationships, FDA offers flexible discrimination, and SRE provides a fundamental environmental envelope model. Finally, an Ensemble Modeling technique (ESMs) was applied to synthesize the collective strength of all individual models, aiming to mitigate their individual weaknesses and produce a single, more accurate and reliable prediction of wind erosion susceptibility. The chosen algorithms represent a broad spectrum of techniques, each contributing unique strengths. The chosen algorithms represent a broad spectrum of techniques, each contributing unique strengths. This comprehensive multi-algorithmic configuration is widely validated in the Middle Eastern and Iranian dust topography as the standard pipeline to handle intricate variable dimensions and non-normal ecological behaviors [6,41]. Some key characteristics of these algorithms are provided below.

Maximum entropy model (MaxEnt)

The Maximum Entropy Model (MaxEnt) is a widely used machine learning algorithm that models ecological distributions based on the principle of maximum entropy, assuming the least biased probability distribution subject to known constraints [42]. In this study, MaxEnt was used to predict the spatial distribution of erosion points by linking ecological data with environmental variables. This model has been successfully applied in biodiversity conservation and environmental modeling, as it is particularly effective when only presence data is available [42,43].

Generalized linear model (GLM)

The Generalized Linear Model (GLM), a parametric modeling approach introduced by Austin et al. (1984) [44], has been extensively applied to model binary outcomes such as the presence or absence of erosion points. In this study, the GLM framework was employed to analyze the relationship between various predictor variables and the occurrence of erosion. This method is well-suited for dealing with non-normal response variables, making it a robust choice for ecological and environmental data analysis [45,46].

Random forest (RF)

Random Forest (RF) is a robust machine learning algorithm that employs an ensemble of decision trees to improve prediction accuracy. By aggregating the results from multiple trees, RF reduces the risk of over-fitting and provides more reliable predictions [47]. In this study, RF was chosen for its effectiveness in both classification and regression tasks, particularly due to its ability to handle large datasets and complex interactions between variables. The method has demonstrated high accuracy in ecological and environmental studies, making it an ideal choice for modeling erosion potential [48,49].

Generalized boosting method (GBM)

The Generalized Boosting Method (GBM) is an ensemble learning technique that combines multiple weak predictive models to create a robust and accurate predictor. GBM applies a random subset of data iteratively, refining its predictions with each iteration by adjusting for errors in previous predictions [48,50]. The method utilizes a sequential boosting approach, where each erosion point is incorporated into the model progressively to enhance predictive accuracy, especially in capturing complex patterns in environmental data [51].

Multivariate adaptive regression splines (MARS)

Multivariate Adaptive Regression Splines (MARS) is a non-parametric regression technique that adapts to the data by dividing the predictor space into regions with different regression functions [52]. This method does not require prior assumptions about the relationship between dependent and independent variables, making it highly flexible and capable of capturing complex interactions in environmental datasets. MARS was particularly useful in this study for analyzing the complex erosion structures, where multiple interacting environmental factors contribute to the erosion potential [52,53].

Artificial neural networks (ANN)

Artificial Neural Networks (ANNs) are a class of machine learning models inspired by the neural architecture of the human brain. ANNs are particularly effective in capturing complex and non-linear relationships between input variables and the target outcome [54,55]. In the context of erosion modeling, ANNs have been utilized to predict the extent of waterway erosion, taking advantage of their flexibility to adapt to diverse environmental conditions and their capacity to learn from experimental data [56,57].

Flexible discriminant analysis (FDA)

Flexible Discriminant Analysis (FDA) is a regression technique that optimizes data separation through the creation of discriminant functions. By using optimal scoring and linear separation, FDA allows for improved classification of erosion-prone regions [58]. This method has been used to develop better predictive models by utilizing multiple regression lines to classify and predict areas with a higher risk of erosion. Its ability to create precise decision boundaries makes it a powerful tool in the field of environmental and geospatial data analysis [59].

Classification tree analysis (CTA)

Classification Tree Analysis (CTA) is a non-parametric method used for predictive modeling and classification tasks. This technique builds a decision tree by recursively partitioning the data into subsets that best separate the different classes [47]. CTA was employed in this study to classify regions based on their erosion susceptibility, helping to identify critical areas for intervention. This method's ability to handle large datasets and reveal complex interactions between variables makes it an invaluable tool in environmental modeling [60,61].

Surface range envelope (SRE)

The Surface Range Envelope (SRE), also known as BIOCLIM, is a statistical technique used to model species distributions based on environmental data [62]. In this study, SRE was employed to predict the distribution of waterway erosion across the study area by correlating environmental variables with erosion occurrence. This model is particularly effective in ecological and environmental studies, as it relies on the ecological principle that species (or in this case, erosion) are constrained by environmental envelopes, such as temperature, moisture, and soil properties [63,64].

Generalized additive model (GAM)

The Generalized Additive Model (GAM) is a non-parametric regression method that allows for flexible modeling of non-linear relationships between predictor variables and the response variable [65]. In this study, GAM was utilized to predict erosion potential by identifying non-linear and non-uniform associations between environmental variables, such as topography and vegetation cover, and the likelihood of erosion events [66]. This method is particularly useful for capturing complex interactions in ecological and geospatial data [67].

Ensemble models (ESMs)

Ensemble Models (ESMs) combine multiple predictive models to enhance the overall accuracy and robustness of predictions. BioMod2, a modeling framework, allows for the training and integration of different algorithms, generating a more reliable outcome through techniques such as weighted averaging [68]. In this study, ESMs were used to integrate multiple models, with each model contributing based on its predictive performance. This technique has been widely used in environmental modeling to improve prediction accuracy and provide more stable results by leveraging the strengths of individual models [69,70].

Estimation of the model performance

The accuracy of the models was evaluated using three commonly used indices: KAPPA, TSS, and ROC [49,55,71–73]. The validity of the different models was assessed using three statistical coefficients. First, the Receiver Operating Characteristic (ROC) curve was employed to evaluate the model's ability to predict the presence or absence of a subject based on related environmental variables [49,72,74]. Second, the True Skill Statistic (TSS) was calculated to assess the performance of presence/absence models [71,74]. TSS can be used to interpret real ecological phenomena. Studies have shown a high correlation between ROC and TSS rates. Therefore, TSS can serve as a suitable alternative to ROC in studies focusing on presence-absence data [75]. Finally, Cohen's Kappa (KAPPA) was used to measure the agreement between two evaluators who have categorized N items into C mutually exclusive categories [76,77]. The values of ROC, KAPPA, and TSS are interpreted as follows: values less than 0.5 indicate poor modeling performance, 0.6–0.5 signify very weak agreement, 0.7–0.6 imply weak agreement, 0.8–0.7 indicate moderate agreement, 0.9–0.8 suggest good agreement, and 0.9–1 indicate very high agreement [78,79].

For a geographic perspective on regions suitable for high wind erosion intensity within the study area, maps depicting wind erosion susceptibility were prepared (Fig 4). The susceptibility maps were generated using machine learning models and are presented on a continuous scale from 0 to 1000. Zero represents the lowest susceptibility, and 1000 indicates the highest susceptibility. These maps were classified in ArcGIS: regions with no erosion susceptibility (0–250), regions with low erosion susceptibility (250–500), regions with moderate erosion susceptibility (500–750), and regions highly susceptible to wind erosion (750–1000) (Table 2).

Results and discussion

In evaluating the high-resolution wind erosion susceptibility mapping across the northeastern sector of Iran, the geomorphological attributes of the Khorasan watershed reveal that a large area of the province, suffering from prolonged aridity and sparse vegetation cover, exhibits bare soil as its primary physical attribute. This kind of surface is known to be prone to water and wind induced soil erosion, whenever rain and wind events occur [32]. The erosion can be particularly pronounced over slopes. The performance of the models is affected by the type of data, extent of the study area, and prevailing environmental conditions [31].

The values of KAPPA, TSS, and ROC, are presented in Table 1. Although all the models exhibited strong predictive performance, as indicated by the ROC, TSS, and Kappa metrics (Table 1), accounting for spatial autocorrelation in model residuals remains a critical consideration in species distribution modeling (SDM). While ensemble methods (ESMs) and algorithms such as Random Forest are generally robust, their predictive outputs can still be influenced by inherent spatial structure within the dataset. Our strategy to mitigate this effect was the spatial thinning of occurrence during the data preparation phase. Among all the models, the Random Forest (RF) model exhibited the highest accuracy for centers with high, medium, and low wind erosion intensities, with values of 87.8%, 77.7%, and 79.8% for KAPPA, TSS, and ROC, respectively. Therefore, the RF model was selected as the preferred model for all three-wind erosion intensity susceptibility assessment and will serve as the basis for subsequent calculations.

Table 1. Accuracy assessment in modeling susceptible locations in the present time.

Wind erosion intensity	H1			H2			H3		
Accuracy assessment	KAPPA	TSS	ROC	KAPPA	TSS	ROC	KAPPA	TSS	ROC
MAXENT	0.73	0.73	0.93	0.62	0.61	0.86	0.63	0.63	0.88
GLM	0.75	0.75	0.93	0.64	0.63	0.87	0.62	0.62	0.87
RF	0.88	0.88	0.97	0.78	0.77	0.94	0.8	0.8	0.95
GBM	0.8	0.8	0.95	0.71	0.69	0.9	0.73	0.72	0.91
MARS	0.76	0.75	0.93	0.62	0.6	0.86	0.62	0.61	0.86
ANN	0.6	0.59	0.81	–	–	–	0.53	0.53	0.82
FDA	0.79	0.78	0.95	0.67	0.66	0.88	0.65	0.64	0.88
CTA	0.78	0.78	0.91	0.67	0.65	0.85	0.68	0.67	0.87
SRE	0.26	0.28	0.64	0.25	0.31	0.65	0.23	0.24	0.62
GAM	0.76	0.75	0.94	0.64	0.63	0.88	0.62	0.62	0.87
ESMs	0.87	0.87	0.98	0.97	0.97	1	0.93	0.93	1

https://doi.org/10.1371/journal.pone.0354288.t001

To ensure the robustness of our model predictions and to evaluate their sensitivity, we implemented several checks. The model development procedure itself incorporated robustness measures; each algorithm was run five times with different random partitions of the data (70% for training, 30% for testing). This allowed us to account for the variability induced by the random selection of training data and to obtain a more stable estimate of model performance. The low standard deviations observed across these five runs for the top-performing models (RF and ESMs) indicated consistent and reliable predictions, reducing concerns about model instability. Furthermore, the ensemble approach (ESMs) inherently mitigates uncertainty by combining multiple individual models, thus producing a more robust and conservative prediction. While a full global sensitivity analysis quantifying the exact influence of each input variable on the prediction variance was beyond the scope of this study, the variable importance analysis presented in Fig 3 provides crucial insights into which factors drive the model's decisions, while the comprehensive geographical outcomes of these predictions are visualized in the susceptibility classifications of Fig 4. This figure illustrates the spatial distribution of model uncertainty associated with the prediction of high-intensity wind erosion (H1) susceptibility in Eastern Iran. The uncertainty was quantified by analyzing the variance among predictions generated by ten distinct machine learning algorithms (including GLM, GBM, RF, MaxEnt, etc.), which were integrated into an Ensemble Model (ESMs) weighted by the True Skill Statistic (TSS). Areas with higher uncertainty (e.g., transition zones between erosion classes) indicate regions where model predictions are less consistent, often due to complex interactions of environmental factors or limitations in training data. Conversely, areas with low uncertainty reflect strong consensus among models, suggesting higher reliability in the susceptibility estimates. This uncertainty assessment provides critical insight for risk managers and policymakers, highlighting priority areas for further data collection and targeted field validation to improve model confidence and support robust land management decisions (Supplementary S4 Fig).

The relative importance of environmental variables differed markedly across the three wind erosion intensity scenarios (H1, H2, H3). The variable importance analysis for high-intensity erosion centers (H1) identified soil properties (Sand, Silt, and Clay content at 0–5 cm depth, and LST), topographic factors (TWI), and climatic variables (BIO4, LST) as the most significant factors governing their geographical distribution (Fig 3a, 3b). The soil texture emerged as the most influential predictors, underscoring the critical role of surface soil properties in sediment availability and erodibility. This is complemented by the significant contribution of Temperature Seasonality (BIO4), which reflects large temperature variations that promote physical weathering and reduce soil moisture, thereby enhancing particle mobility. Land Surface Temperature

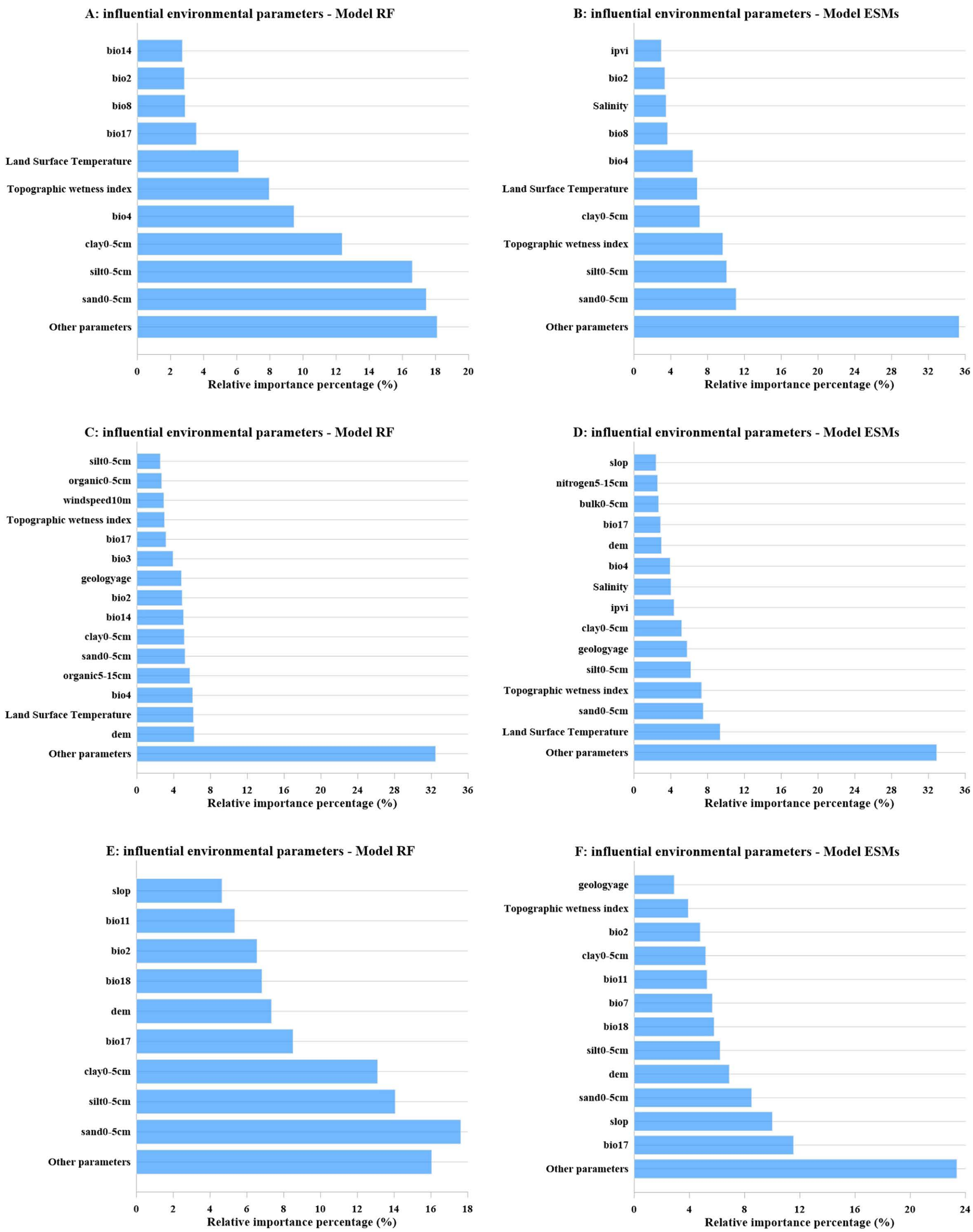

Fig 3. Relative importance percentage of influential environmental parameters on wind erosion intensity in different wind erosion centers. A) Selected Random Forest model for high-intensity wind erosion centers. **B)** Ensemble model for high-intensity wind erosion centers. **C)** Selected Random Forest model for medium-intensity wind erosion centers. **D)** Ensemble model for medium-intensity wind erosion centers. **E)** Selected Random Forest model for low-intensity wind erosion centers. **F)** Ensemble model for low-intensity wind erosion centers.

https://doi.org/10.1371/journal.pone.0354288.g003

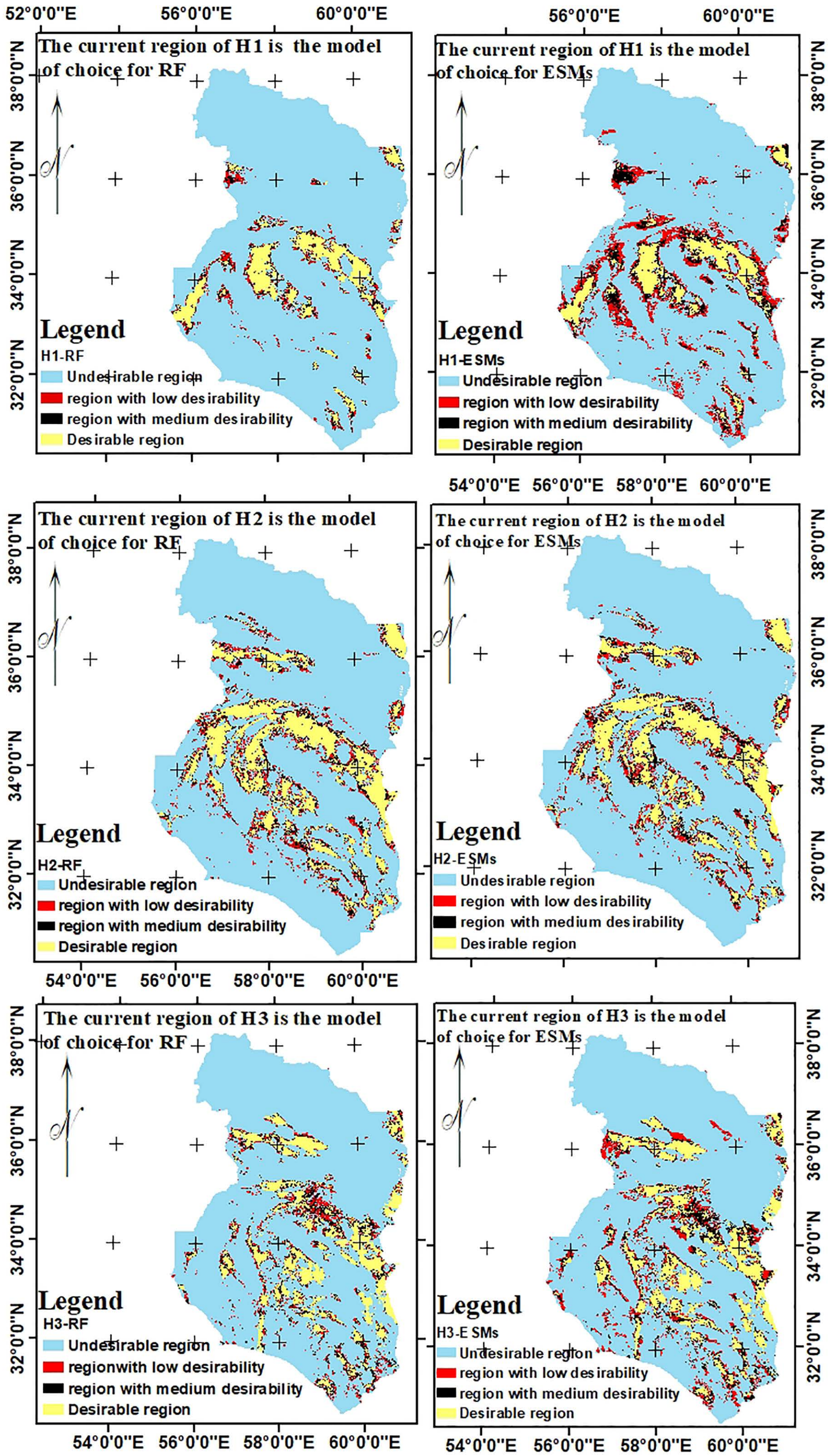

Fig 4. Map of areas with potential for wind erosion in modeling for eastern and northeastern Iran using RF and ESMs models. (All predictive maps and spatial outputs are original, generated by the authors, and formatted under CC BY 4.0 open-access public domain specifications).

https://doi.org/10.1371/journal.pone.0354288.g004

(LST) further indicates surface aridity, a key precondition for erosion initiation. The Topographic Wetness Index (TWI) also features prominently, with lower values (indicating well-drained, upland areas) correlating strongly with high erosion susceptibility due to reduced moisture retention.

For medium-intensity centers (H2), the key predictors expanded to include altitude above sea level, alongside topographic (TWI), climatic (BIO4, BIO14, LST), soil (Sand, Silt, Clay 0–5 cm, LST), and geological factors (Fig 3c, 3d). Here, altitude above sea level joins soil texture and BIO4 as a top predictor, suggesting that elevation-dependent climatic and aerodynamic processes modulate erosion intensity. In contrast, modeling low-intensity erosion centers (H3) highlighted the importance of soil properties, altitude, slope, and specific climatic variables (BIO17, BIO18, BIO2, BIO7) as the primary influencers of their spatial pattern (Fig 3e, 3f). Here, climatic variables such as precipitation of the driest quarter (BIO17) and warmest quarter (BIO18) gain relative importance, highlighting the role of seasonal moisture deficits in predisposing surfaces to lighter erosion. The consistent dominance of soil texture and specific bioclimatic indices across all models confirms their universal relevance in wind erosion dynamics within the study area. These findings are similar to the results of similar studies in other parts of the world [80–82]. These models can be effectively utilized to understand the environmental characteristics of such areas. This aligns with the fact that machine learning models, particularly the RF model, are widely used and are effective for studying potential distribution [31,83,84].

Analysis across all three intensity zones revealed that soil properties, topographic features, climatic conditions, and geological factors are the key drivers determining the spatial distribution of wind erosion centers (Fig 3). The results underscore the predominant role of soil texture, physiography, and climate variables in shaping erosion susceptibility, demonstrating their stronger influence compared to other factors. Variables such as wind speed, the Digital Elevation Model (DEM) itself, soil chemistry, lithology, NDVI, water resources, and salinity indices ranked lower in terms of relative importance and impact. These findings align with previous studies by Boroughani et al. (2021) and Rahmati et al. (2020), who similarly identified soil texture and climate as the most influential factors, with geology and soil chemistry playing a secondary role [85,86].

The spatial predictions generated by the Random Forest (RF) and Ensemble Models (ESMs) revealed significant areas susceptible to wind erosion across the study region (Table 2). The models showed a high degree of consensus in identifying erosion-prone zones, though with variations in the estimated spatial extent. For high-intensity wind erosion (H1), the RF model predicted an area of 26,946 km² (9.13% of the total study area), while the ESMs model estimated a larger susceptible area of 35,075 km² (11.88%). The spatial distribution of these zones indicates the most critical regions requiring immediate mitigation efforts. For medium-intensity erosion (H2), the predicted areas were very similar between models, with RF and ESMs estimating 51,981 km² (17.61%) and 51,648 km² (17.50%) respectively. Concerning low-intensity erosion (H3), the models predicted susceptible areas of 46,176 km² (15.65%) for RF and 50,523 km² (17.12%) for ESMs. In aggregate, these results indicate that approximately 27.03% to 29.70% of the eastern and northeastern regions of Iran face moderate to high wind erosion susceptibility. Without comprehensive land management strategies and targeted conservation policies, these areas are likely to experience accelerated land degradation. Wind erosion causes $650 million in direct damage annually to the agricultural, industrial, and road sectors, and if we add other threatening factors, this figure will be much higher (https://www.iribnews.ir/00GI3r). This environmental challenge carries significant socio-economic implications, potentially exacerbating existing trends of rural-to-urban migration, disrupting agricultural productivity, and undermining local industries, as supported by demographic and economic data from the Statistical Center of Iran.

The susceptibility classes were reclassified into four policy-relevant zones (Table 2) to translate the model outputs into actionable insights for land-use planning and policy formulation. Areas predicted as 'Highly Susceptible' (750–1000) were designated as High-Priority Intervention Zones, requiring immediate and intensive conservation measures such as the establishment of windbreaks, vegetative stabilization, and strict regulation of land use. Regions with 'Moderate Susceptibility' (500–750) were classified as Moderate-Priority Conservation Zones, where practices like controlled grazing, afforestation, and soil moisture conservation are recommended to prevent further degradation. Zones with 'Low Susceptibility' (250–500) were categorized as Low-Priority Monitoring Zones, where the focus should be on monitoring and maintaining

Table 2. Area and percentage of areas with potential for wind erosion in modeling for eastern and northeastern Iran.

Region	Selected model ESMs		Selected model RF		Policy-Relevance Category	Recommended Management Actions
	Area (Km^2)	Area (%)	Area (Km^2)	Area (%)		
**H1: High-intensity wind erosion area**						
Undesirable region (0-250)	231324	79/22	256356	87/79	Stable Zone	Maintain current land management practices; prevent conversion to high-impact activities.
Region with low desirability (250-500)	25596	8/77	8716	2/98	Low-Priority Monitoring Zone	Focus on monitoring land-use changes, maintaining existing vegetation cover, and awareness programs.
Region with medium desirability (500-750)	15679	5/37	6389	2/19	Moderate-Priority Conservation Zone	Implementation of controlled grazing, afforestation projects, water harvesting, and conservation tillage practices.
Desirable region (750-1000)	19400	6/64	20556	7/04	High-Priority Intervention Zone	Urgent implementation of windbreaks, vegetative stabilization, strict land-use regulations, and soil crusting techniques.
**H2: Medium-intensity wind erosion area**						
Undesirable region (0-250)	224059	76/73	224781	76/98	Stable Zone	Maintain current land management practices; prevent conversion to high-impact activities.
Region with low desirability (250-500)	16309	5/59	15255	5/22	Low-Priority Monitoring Zone	Focus on monitoring land-use changes, maintaining existing vegetation cover, and awareness programs.
Region with medium desirability (500-750)	15227	5/21	14200	4/86	Moderate-Priority Conservation Zone	Implementation of controlled grazing, afforestation projects, water harvesting, and conservation tillage practices.
Desirable region (750-1000)	36422	12/47	37781	12/94	High-Priority Intervention Zone	Urgent implementation of windbreaks, vegetative stabilization, strict land-use regulations, and soil crusting techniques.
**H3: Low-intensity wind erosion area**						
Undesirable region (0-250)	221884	75/98	232128	79/49	Stable Zone	Maintain current land management practices; prevent conversion to high-impact activities.
Region with low desirability (250-500)	19622	6/72	13728	4/70	Low-Priority Monitoring Zone	Focus on monitoring land-use changes, maintaining existing vegetation cover, and awareness programs.
Region with medium desirability (500-750)	16909	5/79	13707	4/69	Moderate-Priority Conservation Zone	Implementation of controlled grazing, afforestation projects, water harvesting, and conservation tillage practices.
Desirable region (750-1000)	33614	11/51	32469	11/12	High-Priority Intervention Zone	Urgent implementation of windbreaks, vegetative stabilization, strict land-use regulations, and soil crusting techniques.

https://doi.org/10.1371/journal.pone.0354288.t002

current land conditions to prevent future risk. Finally, areas with 'No Susceptibility' (0–250) were identified as Stable Zones, which can be managed under existing frameworks with a focus on preventing any land-use changes that could induce erosion. This reclassification provides a clear, tiered framework for policymakers and land managers to prioritize interventions and allocate limited resources efficiently, ensuring that mitigation efforts are targeted where they are most needed.

Spatial pattern analysis and interpretation

The susceptibility maps generated by the RF and ESMs models (Fig 4) reveal distinct spatial clustering of high-intensity wind erosion (H1) zones, predominantly concentrated in the central and southern low-lying plains of Khorasan Province. These areas are characterized by a confluence of critical erodibility factors: predominantly sandy-loam soil textures (high

sand content, low cohesion), elevated Land Surface Temperature (LST) indicating surface aridity, low Topographic Wetness Index (TWI) values signifying well-drained surfaces with minimal moisture retention, and high Temperature Seasonality (BIO4) which promotes physical weathering. The convergence of these factors creates an environment highly conducive to particle entrainment and transport by prevailing winds. Conversely, the northern and northeastern high-altitude regions exhibit markedly lower susceptibility, attributed to greater vegetation cover, higher soil moisture (as inferred from lower LST and higher TWI values), and more stable soil aggregates. This clear spatial dichotomy underscores the profound influence of local physiographic and pedo-climatic conditions in controlling erosion patterns. The models successfully delineate not only *where* erosion is most likely but also *why*, by quantitatively linking the spatial outcomes to the dominant environmental drivers identified in the variable importance analysis. This enhanced interpretability is crucial for translating model outputs into targeted land management actions. However, due to the specific features of the studied region and the lack of comprehensive investigation into the distribution of erosion centers and their relation with environmental parameters, further examination is necessary. The findings of Gholami and Mohammadifar (2022) for classifying dust sources in the Middle East using two hybrid DL models (CNN-GRU and DLDL-RF) were consistent with our study [41]. Previous investigations in similar desert environments (e.g., Gholami et al. 2020; Ebrahimi-Khusfi et al., 2021; Boroughani et al. 2021; Momeni et al., 2022; Boroughani et al., 2023) have also mentioned that the RF model is an efficient method for predictive modeling [32,82,85,87,68].

This study investigates the primary environmental drivers of wind erosion by analyzing a suite of influential climatic, physiographic, and pedological (edaphic) factors. A critical climatic variable is Temperature Seasonality (BIO4), where high values indicate significant temperature fluctuations. This seasonality promotes wind erosion through physical weathering, as the repeated expansion and contraction of rocks and soil particles fractures them into finer, more erodible sediments. Furthermore, such temperature variability is frequently coupled with reduced and erratic precipitation, which suppresses vegetation growth and depletes soil moisture, thereby weakening the cohesive forces that bind soil aggregates. Surface aridity is further captured by elevated Land Surface Temperature (LST), which acts as a direct proxy for low soil moisture content. High LST values desiccate the surface soil layer, eliminating moisture-based cohesion and priming particles for transport. However, the kinetic energy required to initiate particle movement is provided solely by wind speed; higher velocities exert greater shear stress on the surface, enabling the entrainment of a larger quantity and size of particles, thus directly controlling erosion intensity. The physiographic context is evaluated through the Topographic Wetness Index (TWI), an indicator of potential moisture accumulation. Areas with low TWI values, typically well-drained arid uplands, were identified in our models as highly susceptible due to their chronically low soil moisture, which reduces cohesion and results in loose, dry surface sediments vulnerable to deflation. Finally, soil texture, a key pedological property, emerged as a fundamental driver. The prevalence of sand particles (0.05–2.0 mm) in the topsoil is a primary contributor, as sandy soils exhibit inherently low cohesion and are easily entrained once wind exceeds threshold velocity. While higher silt and clay content can initially provide stability, these exposed fine particles, when dried, can become a significant source of dust emissions during intense wind events. The interplay between these factors atmospheric energy (wind speed), surface moisture status (LST, TWI), sediment supply (soil texture), and climatic forcing (BIO4) create the optimal conditions for wind erosion processes. However, for a deeper understanding and better recognition, other factors such as human impact, various exploitation, grazing, wildlife, socioeconomic status, and many other factors that directly and indirectly affect wind erosion potential should also be examined.

The growing trend of the dust storm crisis in the east of Iran during the last 30 years has caused an increase in migration. Among the results of these storms, we can point out the disruption in the population and industrial centers in the east of Iran, forcing a shift in labor force away from depleted agriculture and causing the migration of people from villages and small population centers to the surroundings of big cities [6,16].

This environmental challenge carries significant socio-economic implications, potentially exacerbating existing trends of rural-to-urban migration, disrupting agricultural productivity, and undermining local industries. This assertion is supported

by demographic and economic data from the Statistical Center of Iran which report a declining population growth rate and a shift in labor force away from agriculture in the eastern provinces most affected by wind erosion and drought. The projected expansion of erosion-susceptible areas, as modeled in this study, threatens to intensify these adverse socio-economic dynamics.

Model performance

Furthermore, tree-based ensembles like Random Forest are exceptionally robust against multi-resolution data structures, as their hierarchical splitting mechanisms evaluate individual threshold boundaries without requiring spatial data homogeneity. The comparative analysis of the ten machine learning algorithms revealed their predictive performance for wind erosion susceptibility. The superior accuracy of the Random Forest (RF) and Ensemble (ESMs) models, as evidenced by the highest ROC, TSS, and Kappa values across all erosion intensity classes, can be attributed to their inherent algorithmic strengths in handling the complex, non-linear nature of aeolian processes. RF's robustness stems from its ensemble approach of constructing multiple de-correlated decision trees, which effectively mitigates over-fitting a common lacuna with complex environmental datasets. Its ability to model high-dimensional interactions without prior assumptions about variable distribution allowed it to capture the intricate relationships between key drivers like soil texture (sand 0–5 cm), climatic seasonality (BIO4), and topographic moisture (TWI). Conversely, models like Artificial Neural Networks (ANN) and Surface Range Envelope (SRE) under-performed. ANN's performance is often hampered by the requirement for very large training datasets and intricate parameter tuning to avoid over-fitting, which may not have been fully optimized here. The SRE model, being a simplistic environmental envelope technique, fundamentally lacks the capacity to model complex interactions between predictors, leading to poor performance as it only considers the extreme ranges of variables without accounting for their correlations.

Spatial implications

The spatial outputs from the RF and ESMs models are not merely statistical outcomes but carry profound implications for land management and policy. The consensus between these top models in identifying high-intensity erosion zones (H1) in specific geographic clusters provides high confidence for targeting interventions. These areas, predominantly characterized by sandy soil textures, high temperature seasonality, and low topographic wetness, represent hotspots where conservation efforts are required. The spatial prediction of a substantial area (27.03% to 29.70%) under moderate to high susceptibility shows the scale of the challenge. If these projections materialize, the ramifications extend beyond environmental degradation to socio-economic crises, potentially exacerbating rural-to-urban migration patterns and threatening agricultural sustainability, as hinted by the demographic trends from the Statistical Center of Iran. Therefore, the generated susceptibility maps transcend academic exercise; they offer a spatially explicit decision-support framework for prioritizing resource allocation in soil conservation programs, which is crucial for a country like Iran with limited budgets. Future modeling efforts could be enhanced by incorporating dynamic variables such as land use change trajectories and anthropogenic pressure indices to better capture the evolving nature of erosion drivers.

Suggested interventions

In light of the significant extent of land identified as susceptible to wind erosion, our findings necessitate the translation of scientific insight into actionable policy and management strategies. To effectively mitigate the environmental and socio-economic risks outlined, we propose a multi-tiered approach. First, conservation efforts must be prioritized in the high and moderate susceptibility zones (H1 and H2), which collectively encompass approximately 27–30% of the study region. In these critical areas, immediate implementation of mechanical windbreaks, vegetative stabilization using native, drought-resistant species, and surface crusting techniques are essential to reduce sediment mobility. Concurrently, land-use policies must be reformed to restrict high-impact anthropogenic activities; this includes regulating overgrazing,

converting unsustainable agricultural practices to conservation-based tillage, and limiting expansion of irrigation in areas with sandy soil textures and low topographic wetness. Furthermore, sustainable water resource management is a cornerstone of long-term erosion control. Policies promoting water harvesting, efficient irrigation systems, and the restoration of groundwater tables are crucial to maintain soil moisture a key factor in particle cohesion. To support these efforts, a dynamic monitoring system should be established, leveraging remote sensing technologies and the machine learning frameworks developed in this study to provide early warnings and enable adaptive management. Finally, success hinges on an integrated policy framework that fosters coordination between environmental, agricultural, and urban planning agencies. By aligning soil conservation goals with regional development plans, policymakers can not only combat desertification but also safeguard livelihoods, thereby addressing the root causes of migration and economic vulnerability in eastern Iran.

Conclusions

Iran is constantly exposed to dust and sandstorm events. The wind erosion areas that cause the dispersion of dust and sand in Iran require urgent action. Considering the increasing trend of wind erosion areas in Iran, comprehensive maps need to be updated every five years. This is crucial for better quantification of wind erosion and proper implementation of control measures. The first step in reducing wind erosion is to identify vulnerable areas at risk of erosion. This is especially important as the budget for soil protection is limited and the lack of comprehensive management by policymakers in prioritizing areas for erosion control measures could result in irreversible costs. Accurate assessment of wind erosion for large scales in arid and semi-arid environments is a necessity, especially for developing countries like Iran. There is no coordinated method to identify areas prone to wind erosion. Unprecedented exploitation of land along with climate changes and successive droughts have caused the wind erosion areas in the east and northeast of Iran to grow rapidly. In this work, various ML models such as GLM, GBM, CTA, ANN, SRE, FDA, MARS, RF, Maxent and ESMs have been evaluated and the most suitable model has been selected. Based on ROC, TSS and KAPPA, it was found that the performance of RF and ESMs models is significantly higher than other models. The produced maps in RF and ESMs models can not only identify areas susceptible to wind erosion but also pinpoint sensitive and foreseeable areas for the future. Such information plays a crucial role in arid and semi-arid areas, contributing to protective, developmental, and restorative strategies for desertification and ecosystem revival.

In the geographical distribution of wind erosion with selected models of RF and ESMs, high intensity areas (H1) have an area equivalent to 9.23% and 12.01%, and areas with medium intensity (H2) have an area equal to 17.80% and 17.69%, respectively. Low intensity (H3) is introduced in an area equal to 15.81 and 17.30% in the east and northeast of Iran. In general, it can be stated that in the investigated area, if there is no proper and serious planning, we will witness an increase in wind erosion with moderate to high intensity with an area of about 27.03 to 29.7 percent. This will increase immigration and increase unemployment and destroy industries and agriculture. Examining the relative importance of all environmental factors in all three intensities of wind erosion (H1, H2, H3) showed that geological factors (soil texture), physiographic factors (Altitude above sea level and Topographic wetness index and Slope) and climatic factors (BIO4 and BIO17 and BIO18 and BIO2 and BIO7) are significant in the geographical distribution of wind erosion areas in the east and northeast of Iran. This information in the eastern and northeastern regions of Iran can play an effective role in reducing the risks of wind erosion in long-term planning strategies such as:

- Mitigation should focus on H1–H2 zones through windbreaks, vegetative stabilization with native drought-tolerant species, and surface crusting.
- Land-use reforms must limit overgrazing, unsustainable tillage, and irrigation expansion in sandy zones.
- Sustainable water management via harvesting, efficient irrigation, and groundwater restoration is essential to maintain soil moisture.

- A remote-sensing-based monitoring system integrated with ML models can provide early warnings and adaptive management.
- Coordinated policy integration across environmental, agricultural, and urban sectors is crucial to curb desertification and sustain livelihoods in eastern Iran.

Destructive human activities, including unsustainable exploitation of natural resources alongside climate changes and recurrent droughts, have led to the rapid growth of wind erosion centers in eastern and northeastern Iran. Such unsustainable exploitation, without considering the environmental capabilities of natural resources, is a major issue in these regions, eventually leading to the depletion of water, soil, and vegetation. This issue should be addressed at the earliest.

Supporting information

S1 Table. List of predictive variables used in wind erosion susceptibility modeling.
(DOCX)

S1 Fig. Illustrates the Pearson correlation test results of the High Intensity wind erosion areas (H1) for the 90 predictor variables. Negative correlations are represented in red, while positive correlations are shown in blue. The intensity of color and the size of the circles indicate the magnitude of correlation coefficients.
(JPG)

S2 Fig. Illustrates the Pearson correlation test results of the Medium Intensity wind erosion areas (H2) for the 90 predictor variables. Negative correlations are represented in red, while positive correlations are shown in blue. The intensity of color and the size of the circles indicate the magnitude of correlation coefficients.
(JPG)

S3 Fig. Illustrates the Pearson correlation test results of the Low Intensity wind erosion areas (H3) for the 90 predictor variables. Negative correlations are represented in red, while positive correlations are shown in blue. The intensity of color and the size of the circles indicate the magnitude of correlation coefficients.
(JPG)

S4 Fig. The spatial distribution of model uncertainty associated with the prediction of susceptibility to wind erosion in Eastern Iran, for high (H1), medium (H2), and low (H3) intensity areas.
(JPG)

Author contributions

Conceptualization: Tajbakhsh Fakhrabadi Seyed Mohammad, Dericks Shukla.

Data curation: Momeni Damaneh Javad, Memarian Hadi, Komeh Zinatplease.

Formal analysis: Momeni Damaneh Javad, Tajbakhsh Fakhrabadi Seyed Mohammad, Memarian Hadi, Komeh Zinatplease, Dericks Shukla.

Investigation: Momeni Damaneh Javad, Tajbakhsh Fakhrabadi Seyed Mohammad, Memarian Hadi, Komeh Zinatplease, Dericks Shukla.

Methodology: Momeni Damaneh Javad, Tajbakhsh Fakhrabadi Seyed Mohammad.

Project administration: Tajbakhsh Fakhrabadi Seyed Mohammad.

Resources: Tajbakhsh Fakhrabadi Seyed Mohammad, Memarian Hadi, Komeh Zinatplease.

Software: Memarian Hadi.

Supervision: Dericks Shukla.

Validation: Komeh Zinatplease.

Visualization: Momeni Damaneh Javad, Komeh Zinatplease, Dericks Shukla.

Writing – original draft: Momeni Damaneh Javad, Tajbakhsh Fakhrabadi Seyed Mohammad, Memarian Hadi, Komeh Zinatplease.

Writing – review & editing: Dericks Shukla.

References

1. Goudie AS, Middleton NJ. Desert dust in the global system. Berlin, Heidelberg: Springer; 2006. https://doi.org/10.1007/3-540-32355-4_5
2. Shao Y, Wyrwoll K-H, Chappell A, Huang J, Lin Z, McTainsh GH, et al. Dust cycle: an emerging core theme in Earth system science. Aeolian Res. 2011;2(4):181–204. https://doi.org/10.1016/j.aeolia.2011.02.001
3. Safriel UN. Alternative livelihoods for attaining sustainability and security in drylands. In: Coping with global environmental change, disasters and security: threats, challenges, vulnerabilities and risks; 2011 .pp. 835–52. Berlin, Heidelberg: Springer. https://doi.org/10.1007/978-3-642-17776-7_48
4. Goudie AS, Middleton NJ. The changing frequency of dust storms through time. Clim Change. 1992;20(3):197–225. https://doi.org/10.1007/bf00139839
5. Mohammadi M, Gholami H, Mohamadifar A, Song Y, Kaskaoutis D. Enhancing wind erosion hazard assessment: a novel framework combining integrated land susceptibility to wind erosion (ILSWE) index and machine learning algorithms (case study: Saravan area, Southeast Iran). Aeolian Res. 2025 Dec 1;74: 100995. https://doi.org/10.1016/j.aeolia.2025.100995
6. Yousefi R, Wang F, Shaheen A, Ge Q, Kaveh-Firouz A, Kaskaoutis DG, et al. Long-term perspectives on land-use changes and air pollution policies in Iran: a comparative analysis of regional and global patterns in atmospheric PM2.5. J Environ Manage. 2025;375:124346. https://doi.org/10.1016/j.jenvman.2025.124346 PMID: 39892257
7. Stout JE. Diurnal patterns of blowing sand. Earth Surf Processes Landf. 2010;35(3):314–8. https://doi.org/10.1002/esp.1919
8. Middleton NJ. Dust storms in the Middle East. J Arid Environ. 1986;10(2):83–96. https://doi.org/10.1016/s0140-1963(18)31249-7
9. Moulin C, Lambert CE, Dulac F, Dayan U. Control of atmospheric export of dust from North Africa by the North Atlantic Oscillation. Nature. 1997;387(6634):691–4. https://doi.org/10.1038/42679
10. Jish Prakash P, Stenchikov G, Kalenderski S, Osipov S, Bangalath H. The impact of dust storms on the Arabian Peninsula and the Red Sea. Atmos Chem Phys. 2015;15(1):199–222. https://doi.org/10.5194/acp-15-199-2015
11. Suresh K, Singh U, Kumar A, Karri D, Peketi A, Ramaswamy V. Provenance tracing of long-range transported dust over the Northeastern Arabian Sea during the southwest monsoon. Atmos Res. 2021;250:105377. https://doi.org/10.1016/j.atmosres.2020.105377
12. Tyagi G, Babu KN, Solanki HA. Monitoring bio-optical response of coastal waters surrounding the Indian subcontinent to atmospheric dust deposition using satellite data. Environ Sci Pollut Res Int. 2020;27(5):5523–35. https://doi.org/10.1007/s11356-019-07134-2 PMID: 31853851
13. Boroughani M, Zandi R, Pourhashemi S, Gholami H, Kaskaoutis DG. Linking sand/dust storms hotspots and land use over Iran. Atmos Pollut Res. 2025;16(2):102380. https://doi.org/10.1016/j.apr.2024.102380
14. Gholami H, Mohammadifar A, Fitzsimmons KE, Li Y, Kaskaoutis DG. Modeling land susceptibility to wind erosion hazards using LASSO regression and graph convolutional networks. Front Environ Sci. 2023;11. https://doi.org/10.3389/fenvs.2023.1187658
15. UNEP, 2016. Combating sand and dust storms. Resolution adopted by the United Nations Environment Assembly of the United Nations Environment Programme at its second session (UNEA-2), Progress Report/Resolutions, Nairobi, Kenya.
16. Emamian A, Rashki A, Kaskaoutis DG, Gholami A, Opp C, Middleton N. Assessing vegetation restoration potential under different land uses and climatic classes in northeast Iran. Ecol Indic. 2021;122:107325. https://doi.org/10.1016/j.ecolind.2020.107325
17. Gholami H, Jalali M, Rezaei M, Mohamadifar A, Song Y, Li Y, et al. An explainable integrated machine learning model for mapping soil erosion by wind and water in a catchment with three desiccated lakes. Aeolian Res. 2024;67–69:100924. https://doi.org/10.1016/j.aeolia.2024.100924
18. Goudie AS. Desert dust and human health disorders. Environ Int. 2014;63:101–13. https://doi.org/10.1016/j.envint.2013.10.011 PMID: 24275707
19. Boloorani AD, Kazemi Y, Sadeghi A, Shorabeh SN, Argany M. Identification of dust sources using long term satellite and climatic data: a case study of Tigris and Euphrates basin. Atmos Environ. 2020;224:117299. https://doi.org/10.1016/j.atmosenv.2020.117299
20. Hamzeh NH, Kaskaoutis DG, Rashki A, Mohammadpour K. Long-term variability of dust events in Southwestern Iran and its relationship with the drought. Atmosphere. 2021;12(10):1350. https://doi.org/10.3390/atmos12101350
21. Heydarzadeh K, Ruh JB, Vergés J, Hajialibeigi H, Gharabeigli G. Evolution of a structural basin: numerical modelling applied to the Dehdasht Basin, Central Zagros, Iran. J Asian Earth Sci. 2020;187:104088. https://doi.org/10.1016/j.jseaes.2019.104088

22. Bozorgi A, Bozorg-Haddad O, Sima S, Loáiciga HA. Comparison of methods for estimating loss from water storage by evaporation and impacts on reservoir management. Water Environment J. 2020;35(1):218–28. https://doi.org/10.1111/wej.12620
23. Papi R, Attarchi S, Darvishi Boloorani A, Neysani Samany N. Knowledge discovery of Middle East dust sources using Apriori spatial data mining algorithm. Ecol Inform. 2022;72:101867. https://doi.org/10.1016/j.ecoinf.2022.101867
24. Rahmati O, Pourghasemi HR, Melesse AM. Application of GIS-based data driven random forest and maximum entropy models for groundwater potential mapping: a case study at Mehran Region, Iran. Catena. 2016 Feb 1;137: 360–72. https://doi.org/10.1016/j.catena.2015.10.010
25. Sela S, Svoray T, Assouline S. Soil water content variability at the hillslope scale: impact of surface sealing. Water Resour Res. 2012;48(3). https://doi.org/10.1029/2011wr011297
26. Ginoux P, Prospero JM, Gill TE, Hsu NC, Zhao M. Global-scale attribution of anthropogenic and natural dust sources and their emission rates based on MODIS Deep Blue aerosol products. Rev Geophys. 2012;50(3). https://doi.org/10.1029/2012rg000388
27. Wang W, Samat A, Ge Y, Ma L, Tuheti A, Zou S, et al. Quantitative soil wind erosion potential mapping for central Asia using the google earth engine platform. Remote Sens. 2020;12(20):3430. https://doi.org/10.3390/rs12203430
28. Gholami H, Mohamadifar A, Rahimi S, Kaskaoutis DG, Collins AL. Predicting land susceptibility to atmospheric dust emissions in central Iran by combining integrated data mining and a regional climate model. Atmos Pollut Res. 2021;12(4):172–87. https://doi.org/10.1016/j.apr.2021.03.005
29. Rezaei M, Mohammadifar A, Gholami H, Mina M, Riksen MJPM, Ritsema C. Mapping of the wind erodible fraction of soil by bidirectional gated recurrent unit (BiGRU) and bidirectional recurrent neural network (BiRNN) deep learning models. CATENA. 2023;223:106953. https://doi.org/10.1016/j.catena.2023.106953
30. Cao H, Amiraslani F, Liu J, Zhou N. Identification of dust storm source areas in West Asia using multiple environmental datasets. Sci Total Environ. 2015;502:224–35. https://doi.org/10.1016/j.scitotenv.2014.09.025 PMID: 25260168
31. Jafari R, Amiri M, Asgari F, Tarkesh M. Dust source susceptibility mapping based on remote sensing and machine learning techniques. Ecological Informatics. 2022;72:101872. https://doi.org/10.1016/j.ecoinf.2022.101872
32. Boroughani M, Mirchooli F, Hadavifar M, Fiedler S. Mapping land degradation risk due to land susceptibility to dust emission and water erosion. Soil. 2023 Jul 13;9(2):411–23. https://doi.org/10.5194/soil-9-411-2023
33. Choobari OA, Zawar-Reza P, Sturman A. The global distribution of mineral dust and its impacts on the climate system: a review. Atmos Res. 2014;138:152–65. https://doi.org/10.1016/j.atmosres.2013.11.007
34. Damaneh JM, Ahmadi J, Rahmanian S, Sadeghi SMM, Nasiri V, Borz SA. Prediction of wild pistachio ecological niche using machine learning models. Ecological Informatics. 2022;72:101907. https://doi.org/10.1016/j.ecoinf.2022.101907
35. Mosavi Baygi M, Ashraf B. Study of leading to drought of autumn and winter synoptic patterns in Khorasan Razavi Province. J Soil Water Conserv. 2011;18(4):184–167.
36. Boria RA, Olson LE, Goodman SM, Anderson RP. Spatial filtering to reduce sampling bias can improve the performance of ecological niche models. Ecological Modelling. 2014;275:73–7. https://doi.org/10.1016/j.ecolmodel.2013.12.012
37. Zhang X, Yuan Y, Zhu Z, Ma Q, Yu H, Li M, et al. Predicting the distribution of oxytropis ochrocephala bunge in the source region of the yellow river (china) based on UAV sampling data and species distribution model. Remote Sensing. 2021;13(24):5129. https://doi.org/10.3390/rs13245129
38. Momeni Damaneh J, Safdari AA, Azarnejad N, Ghorbani M, Panahi F, Afzali SF, et al. Modeling soil erosion susceptibility using machine learning techniques: Rud-e-Faryab Basin, Iran. Land Degrad Dev. 2025;36(18):6396–409. https://doi.org/10.1002/ldr.70077
39. Thuiller W, Lafourcade B, Engler R, Araújo MB. BIOMOD – a platform for ensemble forecasting of species distributions. Ecography. 2009;32(3):369–73. https://doi.org/10.1111/j.1600-0587.2008.05742.x
40. Momeni DJ, Ahmadi J, Jafarpour CZ. Identification of suitable areas for cultivation of saffron (*Crocus sativus* L.) using artificial intelligence-based models in Khorasan Razavi Province. J Saffron Res. 2023 Oct 23;11(2):328–45. https://doi.org/10.22077/jsr.2024.7137.1231
41. Gholami H, Mohammadifar A. Novel deep learning hybrid models (CNN-GRU and DLDL-RF) for the susceptibility classification of dust sources in the Middle East: a global source. Sci Rep. 2022;12(1):19342. https://doi.org/10.1038/s41598-022-24036-5 PMID: 36369266
42. Phillips SJ, Anderson RP, Schapire RE. Maximum entropy modeling of species geographic distributions. Ecological Modelling. 2006;190(3–4):231–59. https://doi.org/10.1016/j.ecolmodel.2005.03.026
43. Elith J, Phillips SJ, Hastie T, Dudík M, Chee YE, Yates CJ. A statistical explanation of MaxEnt for ecologists. Divers Distrib. 2010;17(1):43–57. https://doi.org/10.1111/j.1472-4642.2010.00725.x
44. Austin MP, Cunningham RB, Fleming PM. New approaches to direct gradient analysis using environmental scalars and statistical curve-fitting procedures. Vegetatio. 1984;55(1):11–27. https://doi.org/10.1007/bf00039976
45. McCullagh P. Generalized linear models.Eur J Oper Res. 1984;16(3):285–92. https://doi.org/10.1016/0377-2217(84)90282-0
46. Venables WN, Ripley BD. Modern applied statistics with S. Springer Science & Business Media; 2013.
47. Breiman L, Friedman J, Olshen R, Stone C. Classification and regression trees. Wadsworth Int. Group. 1984;37(15):237–51.
48. Elith J, Franklin J. Species distribution modeling. In: Encyclopedia of Biodiversity: Second Edition. Elsevier Inc.; 2013. pp. 692–705. https://doi.org/10.1016/B978-0-12-384719-5.00318-X

49. Gupta P, Shukla AK, Shukla DP. Sentinel 2 based burn severity mapping and assessing post-fire impacts on forests and buildings in the Mizoram, a north-eastern Himalayan region. Remote Sens Appl Soc Environ. 2024;36:101279. https://doi.org/10.1016/j.rsase.2024.101279
50. Friedman JH. Greedy function approximation: a gradient boosting machine. Annals of statistics. 2001:1189–232. http://www.jstor.org/stable/2699986
51. De'ath G. Boosted trees for ecological modeling and prediction. Ecology. 2007;88(1):243–51. https://doi.org/10.1890/0012-9658(2007)88[243:btfema]2.0.co;2 PMID: 17489472
52. Friedman JH. Multivariate adaptive regression splines. Ann Statist. 1991;19(1). https://doi.org/10.1214/aos/1176347963
53. Ghasemzadeh A, Ahmed MM. Utilizing naturalistic driving data for in-depth analysis of driver lane-keeping behavior in rain: non-parametric MARS and parametric logistic regression modeling approaches. Transp Res Part C Emerg Technol. 2018 May 1;90: 379–92. https://doi.org/10.1016/j.trc.2018.03.018
54. Harrell Jr FE, Lee KL, Mark DB. Multivariable prognostic models: issues in developing models, evaluating assumptions and adequacy, and measuring and reducing errors. Stat Med. 1996;15(4):361–87. https://doi.org/10.1002/(SICI)1097-0258(19960229)15:4%3C361::AID-SIM168%3E3.0.CO;2-4
55. Gupta A, Aggarwal Y, Aggarwal P. Deep neural network and ANN ensemble for slope stability prediction. Arch Mater Sci Eng. 2022;116(1):14–27. https://doi.org/10.5604/01.3001.0016.0975
56. Cardoso R, de Mattos WD, Marchesini G, Inacio EC, de Figueiredo RM, Rigo SJ. Elevating rotating machinery fault analysis: a multifaceted strategy with FFT, PCA, ANN, and K-means. Comput Electr Eng. 2025;127:110604. https://doi.org/10.1016/j.compeleceng.2025.110604
57. Nielsen RH. Neurocomputing. Reading, MA: Addison-Wesley; 1990.
58. Hastie T, Tibshirani R, Buja A. Flexible discriminant analysis by optimal scoring. J Am Stat Assoc. 1994;89(428):1255–70. https://doi.org/10.1080/01621459.1994.10476866
59. Lehnert LW, Meyer H, Obermeier WA, Silva B, Regeling B, Bendix J. Hyperspectral data analysis in R: the hsdar package. J Stat Softw. 2019 May 27;89: 1–23. https://doi.org/10.18637/jss.v089.i12
60. Loh W. Classification and regression trees. WIREs Data Min Knowl. 2011;1(1):14–23. https://doi.org/10.1002/widm.8
61. Hodroj A, Bouglia R, Ding Y, Zghal M. Machine learning for density prediction and process optimization of 316L stainless steel fabricated by selective laser melting. J Intell Manuf. 2025;37(1):465–79. https://doi.org/10.1007/s10845-024-02554-5
62. Nix HA. A biogeographic analysis of Australian elapid snakes. Atlas of elapid snakes of Australia. 1986;7:4–15.
63. Elith* J, H. Graham* C, P. Anderson R, Dudík M, Ferrier S, Guisan A, et al. Novel methods improve prediction of species' distributions from occurrence data. Ecography. 2006;29(2):129–51. https://doi.org/10.1111/j.2006.0906-7590.04596.x
64. Busby JR. BIOCLIM–a bioclimate analysis and prediction system. Nature conservation: cost effective biological surveys and data analysis. 1991;6:8–9.
65. Hastie T, Tibshirani R. Generalized additive models. Statist Sci. 1986;1(3). https://doi.org/10.1214/ss/1177013604
66. Wood SN. Generalized additive models: an introduction with R. Chapman and hall/CRC; 2017.
67. Wood SN, Pya N, Säfken B. Smoothing parameter and model selection for general smooth models. J Am Stat Assoc. 2016;111(516):1548–63. https://doi.org/10.1080/01621459.2016.1180986
68. Damaneh JM, Tajbakhsh SM, Ahmadi J, Safdari AA. Comparison of species distribution models in determining the habitat landscape of *Pistacia vera* L. specie in Razavi Khorasan province. Water Soil Manag Model. 2022;3(4):77–92. https://doi.org/10.22098/mmws.2022.11698.1160
69. Anderson RP, Lew D, Peterson AT. Evaluating predictive models of species' distributions: criteria for selecting optimal models. Ecological Modelling. 2003;162(3):211–32. https://doi.org/10.1016/s0304-3800(02)00349-6
70. Latif QS, Saab VA, Dudley JG, Hollenbeck JP. Ensemble modeling to predict habitat suitability for a large-scale disturbance specialist. Ecol Evol. 2013;3(13):4348–64. https://doi.org/10.1002/ece3.790 PMID: 24340177
71. Singh A, Chhetri NK, Nitesh, Gupta SK, Shukla DP. Strategies for sampling pseudo-absences of landslide locations for landslide susceptibility mapping in complex mountainous terrain of Northwest Himalaya. Bull Eng Geol Environ. 2023;82(8). https://doi.org/10.1007/s10064-023-03333-x
72. Singh A, Dhiman N, K C N, Shukla DP. Improving ML-based landslide susceptibility using ensemble method for sample selection: a case study of Kangra district in Himachal Pradesh, India. Environ Sci Pollut Res Int. 2025;32(59):31180–203. https://doi.org/10.1007/s11356-024-34726-4 PMID: 39223412
73. Gupta P, Shukla DP. Demi-decadal land use land cover change analysis of Mizoram, India, with topographic correction using machine learning algorithm. Environ Sci Pollut Res Int. 2024;31(21):30569–91. https://doi.org/10.1007/s11356-024-33094-3 PMID: 38609681
74. Gupta SK, Shukla DP. Handling data imbalance in machine learning based landslide susceptibility mapping: a case study of Mandakini River Basin, North-Western Himalayas. Landslides. 2023;20(5):933–49. https://doi.org/10.1007/s10346-022-01998-1
75. Walther G-R, Post E, Convey P, Menzel A, Parmesan C, Beebee TJC, et al. Ecological responses to recent climate change. Nature. 2002;416(6879):389–95. https://doi.org/10.1038/416389a PMID: 11919621
76. Smeeton NC. Early history of the kappa statistic. Biometrics. 1985;41: 795.
77. Galton F. Finger prints. London and New York: MacMillan; 1892.

78. Yi YJ, Cheng X, Yang ZF, Zhang SH. Maxent modeling for predicting the potential distribution of endangered medicinal plant (H. riparia Lour) in Yunnan, China. Ecol Eng. 2016 Jul 1;92: 260–9. https://doi.org/10.1016/j.ecoleng.2016.04.010
79. Swets JA. Measuring the accuracy of diagnostic systems. Science. 1988;240(4857):1285–93.
80. Genuer R, Poggi J-M, Tuleau-Malot C, Villa-Vialaneix N. Random forests for big data. Big Data Res. 2017;9:28–46. https://doi.org/10.1016/j.bdr.2017.07.003
81. Giang PQ, Trang NT, Anh TT, Binh NT. Prediction of economic loss of rice production due to flood inundation under climate change impacts using a modeling approach: a case study in Ha Tinh Province, Vietnam. Climate Change. 2020;6(21):52–63.
82. Ebrahimi-Khusfi Z, Nafarzadegan AR, Dargahian F. Predicting the number of dusty days around the desert wetlands in southeastern Iran using feature selection and machine learning techniques. Ecol Indic. 2021;125:107499. https://doi.org/10.1016/j.ecolind.2021.107499
83. Golestaneh SR, Karampour F, Farrar N. Introduction of the destructive agents affecting wild almond Amygdalus scoparia forests in Koh-Siah Dashti area in Bushehr province. Iran J For Range Prot Res. 2012;10(2):153–64. https://doi.org/10.22092/ijfrpr.2012.11146
84. Avand M, Mohammadi M, Mirchooli F, Kavian A, Tiefenbacher JP. A new approach for smart soil erosion modeling: integration of empirical and machine-learning models. Environ Model Assess. 2022;28(1):145–60. https://doi.org/10.1007/s10666-022-09858-x
85. Boroughani M, Pourhashemi S, Gholami H, Kaskaoutis DG. Predicting of dust storm source by combining remote sensing, statistic-based predictive models and game theory in the Sistan watershed, southwestern Asia. J Arid Land. 2021;13(11):1103–21. https://doi.org/10.1007/s40333-021-0023-3
86. Rahmati O, Mohammadi F, Ghiasi SS, Tiefenbacher J, Moghaddam DD, Coulon F, et al. Identifying sources of dust aerosol using a new framework based on remote sensing and modelling. Sci Total Environ. 2020;737:139508. https://doi.org/10.1016/j.scitotenv.2020.139508 PMID: 32531509
87. Gholami H, Mohamadifar A, Collins AL. Spatial mapping of the provenance of storm dust: application of data mining and ensemble modelling. Atmos Res. 2020;233:104716. https://doi.org/10.1016/j.atmosres.2019.104716
