# Supplementary material for: Correction: Modeling wind erosion susceptibility of Eastern Iran using machine learning
Source: PLoS One. 2026 Oct 6;21(10):e0360012. doi: 10.1371/journal.pone.0360012 (PMC13641335; doi:10.1371/journal.pone.0360012)
Supplement: Supplementary file 2 [file pone.0360012.s002.pdf]

PLOS One

Correction: Modeling wind erosion susceptibility of Eastern Iran using machine learning

--Manuscript Draft--

**Manuscript Number:**	
**Article Type:**	Correction
**Corresponding Author:**	Emaan Basat<br>PLOS: Public Library of Science<br>San Francisco, UNITED KINGDOM OF GREAT BRITAIN AND NORTHERN IRELAND
**First Author:**	Emaan Basat
**Order of Authors:**	Emaan Basat

Powered by Editorial Manager® and ProduXion Manager® from Aries Systems Corporation
